# Pathogen Enzyme-Mediated Alkoxyamine Homolysis as a Killing Mechanism of *Aspergillus fumigatus*

**DOI:** 10.3390/jof11070503

**Published:** 2025-07-04

**Authors:** Marion Filliâtre, Pierre Voisin, Seda Seren, Ines Kelkoul, Olivier Glehen, Philippe Mellet, Sophie Thétiot-Laurent, Jean Menotti, Sylvain R. A. Marque, Gérard Audran, Abderrazzak Bentaher

**Affiliations:** 1 Aix-Marseille University, CNRS, ICR-UMR 7273, 13007 Marseille, France; marion.filliatre@univ-amu.fr (M.F.); sophie.thetiot-laurent@univ-amu.fr (S.T.-L.); 2 Bordeaux University, CNRS, MRBS-UMR 5536, 33076 Bordeaux, France; pierre.voisin@rmsb.u-bordeaux.fr (P.V.); sseren@rmsb.u-bordeaux.fr (S.S.); i.kelkoul@u-bordeaux.fr (I.K.); 3 Claude Bernard Lyon 1 University, CICLY-UR3738 and Department of Digestive Surgery, Lyon Hospital, 69000 Lyon, France; olivier.glehen@chu-lyon.fr; 4 Claude Bernard Lyon 1 University, CICLY-UR3738 and Department of Parasitology and Mycology, La Croix-Rousse Hospital, 69004 Lyon, France; jean.menotti@univ-lyon1.fr; 5 Claude Bernard Lyon 1 University, CICLY-UR3738 and INSERM, 69495 Pierre-Bénite, France

**Keywords:** alkoxyamine, radical chemistry, *Aspergillus fumigatus*, dipeptidyl peptidase

## Abstract

The emergence of antifungal-resistant *Aspergillus fumigatus (A. fumigatus)* became a serious public health concern, underscoring the need for new effective antifungal agents. Here, we present a strategy based on the in situ generation of radical species that are toxic to the pathogen. The synthesis of an alkoxyamine linked to a peptide substrate recognized by *A. fumigatus*-secreted dipeptidyl peptidase is described. Kinetic experiments show a stable prodrug prior to enzymatic activation. Ensuing peptide cleavage and spontaneous homolysis resulted in the generation of a stable nitroxide and a reactive alkyl radical moiety. Next, the exposure of *A. fumigatus* spores to the prodrug lead to pathogen growth inhibition in a compound concentration-dependent fashion (e.g., 42% inhibition at 10 µg/L). Importantly, the designed alkoxyamine inhibited not only the growth of a clinical voriconazole-susceptible *A. fumigatus* strain, but also the growth of a strain resistant to this azole. To determine the antifungal importance of the reactive alkyl radical, its substitution with a non-radical structure did not prevent *A. fumigatus* growth. Furthermore, the introduction of succinic group in the peptide substrate resulted in the loss of alkoxyamine antifungal properties. Our work reports a novel chemical strategy for antifungal therapy against *A. fumigatus* based on the pathogen enzyme-mediated generation of toxic radicals. Significantly, these findings are timely since they could overcome the emerged resistance to conventional drugs that are known to target defined pathogen biologic mechanisms such as ergosterol synthesis.

## 1. Introduction

*Aspergillus fumigatus* (*A. fumigatus*) is a “common mold” that can lead to serious health issues in vulnerable populations, especially individuals with weakened immune systems or underlying respiratory conditions. Indeed, more than 300 million people worldwide including both immunocompetent and immunocompromised patients suffer from a serious fungal infection [1]. And patients with immune deficiencies (e.g., defects in lymphocytes, macrophages, or neutrophils) are particularly exposed to invasive *Aspergillus* infections [2]. Clinically, *A. fumigatus* has been reported to cause invasive and chronic infections that exacerbate chronic obstructive pulmonary disease, cystic fibrosis, hematology malignancies, or respiratory viral infections such as COVID-19 [3]. Interestingly, *A. fumigatus* belongs to the critical priority fungal group that has been recently recognized by the WHO [4].

In recent years, *A. fumigatus* has indeed become a serious concern in the emergence of antifungal resistance, which complicates treatments of the associated infections. Thus, resistance to common antifungal agents, namely, azoles (e.g., voriconazole), that usually represent the first-line treatment for invasive aspergillosis has been increasingly reported [5]. These resistance mechanisms involve mutations in the target enzyme (lanosterol demethylase) that azoles inhibit, the increased expression of efflux pumps that expel the drug from the cell, or biofilm formation that protects the fungi from antifungal agents [6]. Another concern relates to the side effects of these antifungals, in that these drugs can lead to gastrointestinal symptoms such as nausea/vomiting, headaches/dizziness, and potentially liver damage [7]. Together, these observations underscore the need for new antifungal agents/systems to combat effectively resistant strains as well as to alleviate their associated side effects. The antimicrobial capacity of oxidants such as reactive oxygen species has been largely documented [8]. But, because of their collateral damage to the host due to their high reactivity and lack of selectivity, these species cannot be directly used. In this work, we report an antifungal alternative based on the generation of highly reactive oxygen species in the vicinity of *A. fumigatus*. It concerns the development of new families of alkoxyamines. Of note, the biological properties and applications of alkoxyamines were (and continue to be) explored in various fields including medicinal chemistry, drug delivery, and diagnostics. With respect to the biological properties, alkoxyamines are stable in aqueous phases and can exhibit redox activity following their homolytic cleavage (e.g., this work). Importantly, they can also be functionalized for defined applications. Indeed, we and others have reported the synthesis of alkoxyamines with theranostic properties against cancerous cells [9,10]. Others applications also included their use as parasitic drugs (e.g., malaria, *Schistosomiasis*) [11,12].

**Scheme 1:**
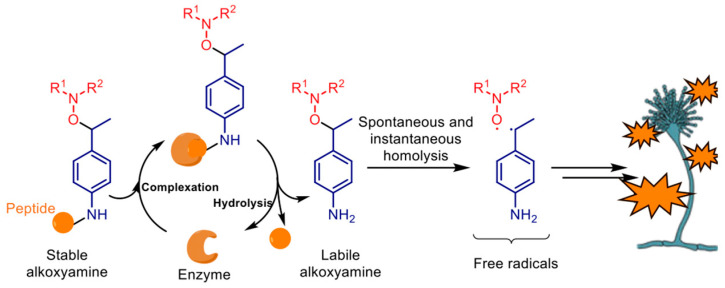
Concept of protease-mediated activation of alkoxyamine prodrugs.

The originality of our approach involves the generation of stable alkoxyamines that are biologically activated by the pathogen’s own enzymatic activity to generate a labile alkoxyamine. The latter spontaneously homolyzes into deadly radical species. To our knowledge, this is the first report that highlights a novel chemical strategy for antifungal therapy against *A. fumigatus* that bypasses the resistance mechanisms developed by *A. fumigatus*, in particular, against azole-based drugs. This concept is as follows (Scheme 1).

## 2. Materials and Methods

### 2.1. Alkoxyamine Synthesis

All chemicals were reagent-grade and were used as-received for the alkoxyamine preparation. Alkoxyamine synthesis, purification, and monitoring steps were carried out following our routinely used methods that were previously reported [13]. The purity of final compounds was >95% as assessed by NMR and/or HPLC, as described elsewhere [14]. A series of alkoxyamines were prepared, and those that exhibited biological activities as well as their corresponding controls are depicted in Supplementary Figure S1. Briefly, prodrug synthesis involves a cascade of coupling steps starting with a commercially available 4-vinylaniline. Where appropriate, the succinic group was coupled with synthesized molecules. In parallel experiments, the addition of nitroxide group was purposely omitted. The detailed information about the synthesis of the prodrugs including NMR spectra and HRMS chromatographs are reported in Supplementary Figure S2.

### 2.2. Stability of Prodrugs

Homolysis constants were measured by Electronic Paramagnetic Resonance (EPR) on a EMX Bruker spectrometer, with O_2_ as the alkyl radical scavenger, as previously reported [15]. Thermic activation led to the liberation of nitroxide: (2,2,6,6-tetramethylpiperidin-1-yl)oxyl (TEMPO). The details of the kinetic experiments in aqueous or solvent phases and the values of homolysis rate constants (kd), activation energies (Ea), and half-life times (t1/2) are reported in Supplementary Information and Data (see below).

### 2.3. Kinetics of Prodrug Enzymatic Activation

The enzymatic hydrolysis of the substrate peptide and the subsequent activation of the alkoxyamine moiety were tested as follows. The prodrug (1 mM) was incubated alone in the presence of dipeptidyl peptidase, fibroblast activation protein (FAP) (3 × 10^−7^ M) (Acrobiosystem) in HEPES buffer (50 mM pH 7.4), 0.15 M NaCl, and 0.5 mM Hemin (Santa Cruz Chemicals) at 37 °C. Since the alkoxyamine instantly homolyzed, upon enzymatic activation, into a stable nitroxide radical and a very unstable and reactive alkyl radical, kinetic hydrolysis was then recorded by following the concentration of generated nitroxide using EPR over time as previously reported [15].

### 2.4. Fungal Cultures

Ku80 azole-susceptible strain and FC240 azole-resistant strain were kindly provided by Prof. J.-P. Latge and Dr E. Fréalle (Lille University Hospitals, Lille, France). The latter strain harbors a 34 bp tandem repeat mutation (TR34) in the cyp51A gene promoter combined with a substitution in codon L98H. The strains of *A. fumigatus* were used for spore preparation as previously described [16,17]. Briefly, spores were grown from frozen stocks on Sabouraud + Chloramphenicol agar tubes (BioMérieux, Marcy l’Etoile, France) at 37 °C for 7 days. Next, conidia were collected by scraping and suspending in PBS containing 0.01% Tween 20 (Sigma-Aldrich^®^, St. Louis, MO, USA). Spore counting was performed using a Kova^®^ hemacytometer, and aliquots of 10^6^ spores were prepared.

### 2.5. Exposure of A. fumigatus Spores to Alkoxyamines

*A. fumigatus* spores were exposed to various concentrations of alkoxyamines (1, 10, 100, and 1000 µM), with each concentration in triplicate. Controls were grown alone (without alkoxyamine treatment) or exposed to the antifungal voriconazole under the same experimental conditions. Briefly, the reactions were performed in well plates in a total volume of 250 μL RPMI medium. At the end of the incubation time period (48 h), the optical densities (OD_600_ nm) of scrapped cultures were recorded in one set of the reactions using a spectrophotometer (Thermo Fisher Scientific, Illkirch, France). In the other set of reactions, the plates were viewed with optical microscopy at a magnification of 200X and photographed, and the areas of fungal lysis were determined. Experiments were repeated at least three times.

### 2.6. Exposure of Epithelial Cells to Alkoxyamine Va

Mouse alveolar cell line MLE15 (kindly provided by J. Wittset, University of Cincinati, Cincinati, OH, USA) were cultured on plates up to confluence in RPMI 1640 culture media as previously described [18,19]. Prior to any treatment, cells were washed three times with sterile phosphate-buffered saline. Next, cell monolayers were cultured alone or in the presence of designated concentrations of alkoxyamine Va (10, 100, and 1000 µM) for 24 h. At the end of treatment time, the plates were viewed with optical microscopy at a magnification of 200X, and the representative images of each condition of the experiments were captured in a blinded manner.

### 2.7. Statistics

Statistical tests were performed using the Prism version 8.4.2 software (GraphPad Software, La Jolla, CA, USA), and data were expressed as mean and standard deviation (±). To compare the difference between the 2 groups, normality was verified using the Shapiro–Wilk test; then, data were analyzed with Student’s *t* test. A *p* < 0.05 was considered statistically significant.

## 3. Results

### 3.1. Prodrug Synthesis

**Scheme 2:**
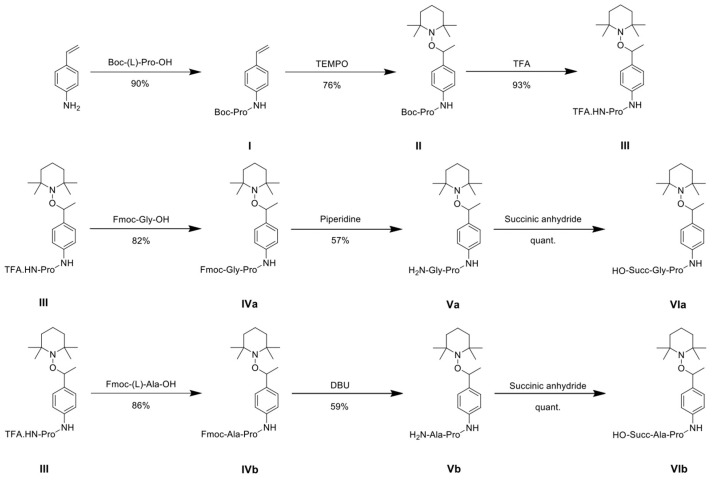
Synthesis of prodrugs Gly-Pro (Va), Ala-Pro (Vb), Succ-Gly-Pro (VIa), and Succ-Ala-Pro (VIb).

The synthesis of Va/Vb and VIa/VIb was performed in five and six steps, respectively (See Scheme 2 and Supplementary Figure S1A,B). Thus, product **I** (linked 4-vinylaniline and Boc-(L)-Pro-OH) is coupled with TEMPO by the action of Jacobsen’s catalyst to give product **II**. Deprotection of the latter with trifluoroacetic acid followed by bio-conjugation between product **III** with either Fmoc-Gly-OH or Fmoc-(L)-Ala-OH in DCM with standard procedure (EDCI, HOBt) generated the products IVa and IVb. Next, the amines of products IVa and IVb were deprotected to give the products Va and Vb (Supplementary Figure S1A). Finally, the products VIa and VIb were synthesized by the reaction of products Va and Vb with succinic anhydride in DCM, and they served as controls (Supplementary Figure S1B). Also, controls included a product where the nitroxide was omitted during the synthesis (Supplementary Figure S1C).

### 3.2. Stability of Prodrugs

The results show that all synthesized alkoxyamines are stable in both organic (*t*-BuPh) and aqueous environments. Indeed, the activation energies of these alkoxyamines range from 121 to 140 kJ/mol with half-life times longer than 10 days (Supplementary Table S1). Moreover, the values were measured for a mixture of the two diastereoisomers. The data show the strictly monoexponential growth of nitroxide, meaning that both diastereoisomers exhibit identical kd values (Supplementary Figure S3). The solvent effect (i.e., *t*-BuPh) versus H_2_O/1-PrOH (99:1) was also investigated. As shown in Supplementary Table S1, the stability of the lead molecule Va, for instance, was altered, that is, t1/2 = 1537 days in t-BuPh and t1/2 = 308 days in H2O/1-PrOH (99:1). Nevertheless, in our experimental conditions of antifungal assays (i.e., 2 days of exposure time), Va can clearly be considered as stable with less than 2% decomposition. Similar findings were made for other molecules such as Vb, Via, and VIb. Overall, there is no stability concern for these molecules with respect to the subsequent biological studies.

### 3.3. Enzymatic Activation of Prodrug

**Figure 1:**
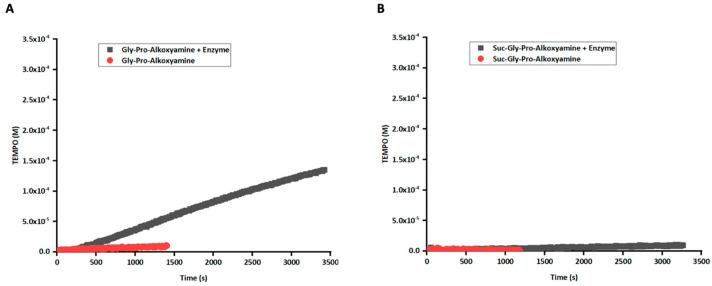
**Enzymatic activation followed by the homolysis of the alkoxyamine.** (**A**) The activation of Gly-Pro-alkoxyamines over time as monitored by TEMPO release using EPR in the presence (black curve) or absence (red curve) of the enzyme, dipeptidyl peptidase IV. (**B**) The presence of the succinic group at the *N*-terminal end of the peptide blocked the enzyme activity and, hence, homolysis.

Figure 1A shows that the enzyme activates Gly-Pro-alkoxyamines, while they remain stable when untreated. Similar data were obtained for Ala-Pro-alkoxyamines (Supplementary Figure S4A). As expected, the presence of the succinic group at the *N*-terminal end of the peptide blocked the dipeptidyl activity for both prodrugs (Figure 1B and Supplementary Figure S4B). Thus, the succinylated prodrugs could be used as negative controls in the *A. fumigatus* growth experiments. All subsequent experiments were carried out with the products Va, VIa, and VIc.

### 3.4. Killing of A. fumigatus by Alkoxyamines

**Figure 2:**
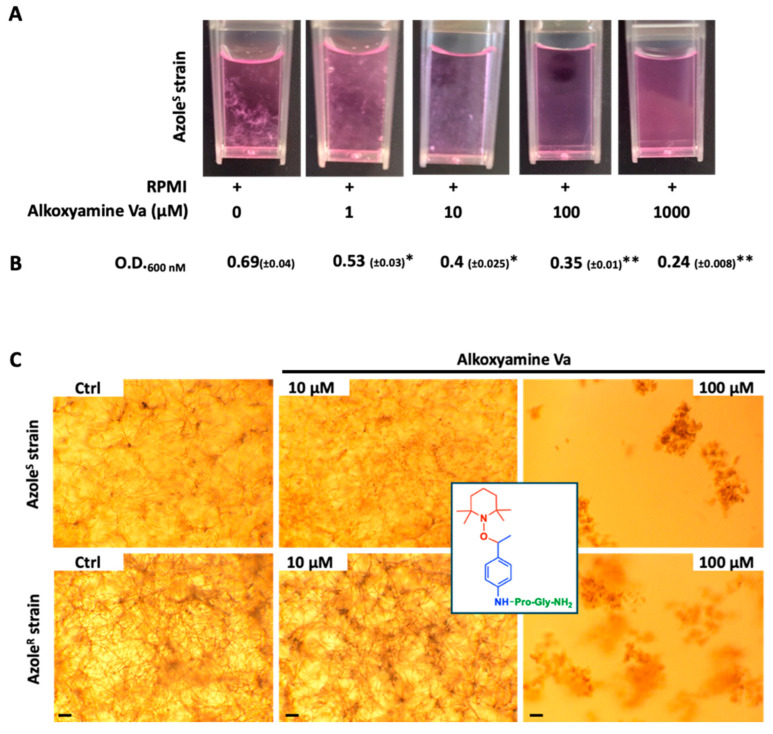
**The killing of *A. fumigatus* by alkoxyamines.** (**A**) The representative micrographs of cultured azole-sensitive *A. fumigatus* alone or in the presence of the prodrug Va. Untreated fungi grew normally as demonstrated by the marked formation of hyphae (lane 0). The addition of Va resulted in the progressive growth inhibition as judged by the gradual and complete loss of hyphae formation (lanes 1 to 1000). (**B**) The optical density values of the cultures following the incubation of *A. fumigatus* alone or with the prodrug Va. The ODs of the reactions were recorded and reflected fungal growth. Untreated cultures grew normally as demonstrated by the increased OD values that corroborated hyphae formation (lane 0). The addition of Va resulted, however, in the progressive decrease in OD coinciding with hyphae loss (lanes 1 to 1000). The OD values represent the mean of three experiments (*, *p* < 0.05 for *A. fumigatus* + Va at 1 and 10 μM versus control; **, *p* < 0.01 for *A. fumigatus* + Va at 100 and 1000 μM versus control, respectively, Student’s *t* test). (**C**) The representative micrographs of cultured azole-resistant *A. fumigatus* in the presence of alkoxyamine Va. Unlike untreated fungi, the addition of Va resulted in the progressive growth inhibition as judged by the gradual and complete loss of hyphae formation (panels 10 and 100 μM). Bar = 8 μm.

To assess the role of our alkoxyamines in mediating the killing of *A. fumigatus*, we used a clinical strain of *A. fumigatus*. As shown in Figure 2A, the product Va mediated the inhibition of *A. fumigatus* growth in a compound concentration-dependent fashion. A noticeable decrease in fungal hyphae could be observed at concentrations as low as 10 μM, and this decrease became marked starting 100 μM. These observations were further corroborated by optical density values (Figure 2B).

Next, the effect of our prodrug was assessed on two clinical strains of *A. fumigatus,* sensitive (Azole^S^) and resistant (Azole^R^) to azole. Interestingly, the alkoxyamine Va inhibited the growth of both strains, as could be judged by the gradual loss of hyphae starting at 10 μM of Va and the numerous areas of fungal lysis at 100 μM of the prodrug when compared to controls (Figure 2A). As a positive control, we confirmed that these strains are indeed susceptible to the antifungal voriconazole, and in accordance with the literature, the MIC of this antifungal against the susceptible and resistant strains was 0.25 mg/L and 4 mg/L, respectively.

### 3.5. Antifungal Efficiency of Alkoxyamines Requires Both Defined Peptide Substrate and Radical Structure

**Figure 3:**
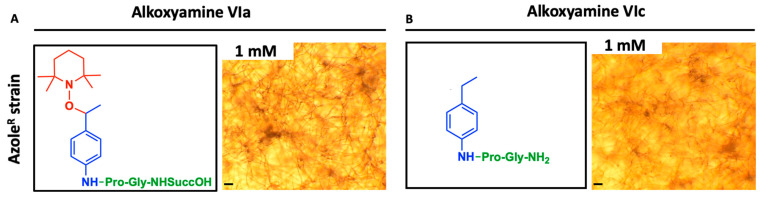
**Alkoxyamines require both defined peptide substrate and radical structure to kill *A. fumigatus*.** The representative micrographs of cultured azole-resistant *A. fumigatus* in the presence of modified prodrugs. (**A**) The addition of the succinic group amino acid peptide resulted in the alkoxyamine antifungal properties since azole-resistant *fumigatus* grew normally. (**B**) The omission of nitroxide in the prodrug led to the loss of the antifungal activity of the latter. Note the normal growth of azole-resistant *A. fumigatus*. Bar = 8 μm.

To demonstrate the contribution of the peptide substrate to the activation of the alkoxyamines, a succinic group was grafted in product Va. Our data clearly shows that *A.* of Azole^R^ *fumigatus* grew normally despite the use of high concentration of this new product (Figure 3A). Next, to determine the importance of the alkyl radical in alkoxyamine-mediated antifungal activity, we synthetized a control molecule with no nitroxide part (product VIc). As shown in Figure 3B, even 1 mM of this product did not impact the growth of Azole^R^ *A. fumigatus*.

### 3.6. Alteration of Epithelial Cell Monolayer Following Exposure to Alkoxyamine Va

Next, to examine the impact of the prodrug on the integrity of epithelial cell monolayer, similar concentrations of the alkoxyamine Va were added to cultured cells. Unlike control cells with flat polygonal morphology (Supplementary Figure S5A), treated cells lost their integrity. Indeed, as the concentrations of the prodrugs increased, the cells detached from each other, displayed rounded morphology, and were floating (Supplementary Figure S5B–D).

## 4. Discussion

In this work, we report an alkoxyamine (AA)-based prodrug that exhibits antifungal properties. Specifically, we designed a stable proform of AA-based chemical structure composed of two moieties: a peptide substrate recognized by a dipeptidyl peptidase 4 (Dpp 4) secreted by *A. fumigatus* that serves as the activating system followed by a labile AA. The latter is made of a nitroxyl fragment, which weakens the C-ON bond of the compound and modulates its hydrophilic/lipophilic properties, and an alkyl fragment. Upon the enzymatic cleavage of the peptide substrate, a spontaneous and instantaneous homolysis frees up a toxic radical that eradicates the fungus. The efficacy of this design was further confirmed since changes in the activation peptide sequence or in the alkyl structure resulted in the loss of the molecule’s antimicrobial properties and normal fungus growth. Importantly, the antifungal propensity of our AA was maintained independently of the fungus phenotype. Indeed, the growth of both azole-sensitive and -resistant *A. fumigatus* strains was clearly hampered in the presence of activated AA.

With respect to the former moiety (activation system), we chose a peptide substrate that is recognized by Dpp4 and served as the prodrug-activating enzyme. In fact, Dpp4 is secreted by *A. fumigatus* and may affect the overall physiology of the host in the setting of infection, i.e., the enzyme could play a role in the colonization step of host tissues [20].

Such design allows the vector system to be hydrolyzed “on demand” in the vicinity of the pathogen (i.e., near the process of enzymatic hydrolysis), thereby releasing a highly labile alkoxyamine. The latter instantaneously and spontaneously “split” into nitroxide (stable molecule) and a toxic alkyl radical with antimicrobial activity against the fungus of interest. Of note, the reactivity of the radicals is so fast that there should be insignificant (or no) radical travel concerns.

While we clearly provide a proof for our concept, careful attention must be paid to the benefit/risk ratio of such designed molecules. Indeed, the Dpp4 enzyme in *A. fumigatus* and human DPP IV share significant homology both in sequence and functional characteristics such as substrate specificity/recognition [20]. This similarity underscores the potential of human DPP IV to recognize and activate the prodrug, leading to increased radical generation and, hence, cytotoxicity toward mammalian cells. This suggests that, while this drug lacks selectivity/specificity towards the pathogen, it could potentially harm the host. This is, indeed, supported by the death of the epithelial cells upon exposure to these drugs. Exploratory studies are ongoing to identify secreted enzyme(s) whose expression is more specific to *A. fumigatus* with less cross-reactivity with human enzymes.

As mentioned above, different antifungal resistance mechanisms developed by *A. fumigatus* have been reported [5,6,21]. These include, for example, mutations in the gene encoding lanosterol 14α-demethylase, an enzyme involved in ergosterol synthesis, that reduce azole binding, rendering the fungus azole-resistant. Another resistance mechanism involves the pathogen ability to alter its membrane composition, preventing the antifungal Amphotericin B from targeting ergosterol, a crucial component of the fungal cell membrane. Accordingly, the originality of our strategic approach is several folds. It resides in the generation of toxic radicals that overcome the antifungal resistance mechanisms. The design is highly versatile as the activation substrate can be adapted to the pathogen-derived enzyme of interest. Thus, this moiety could be organic (amino acid, sugar) or inorganic (metal ions) depending on the activating enzyme of choice. Moreover, the applicability of this approach could be easily extended not only to other pathogenic fungi, but also to other microbes such as bacteria in addition to those recently reported [14,22]. A reassuring point based on the published reports in the literature is that alkoxyamines show promising biological safety profiles. In fact, in vitro, in vivo, and occupational exposure studies support the potential of these drugs for clinical and industrial applications, especially when they can be functionalized [9,23]. Nonetheless, research must be pursued to better our understanding of their long-term safety and efficacy. It must be emphasized that, while the proof of our concept is evidenced, further studies are warranted to address host-related issues such as delivery and release systems (e.g., inhalation, topical, and on-demand), as well as pharmacokinetic and pharmacodynamic properties of the drug. Notwithstanding, the development of this antimicrobial system is timely as it addresses the worldwide concern of antimicrobial resistance.

## Data Availability

Data is contained within the article or Supplementary Materials.

## Supplementary Materials

The following supporting information can be downloaded at: https://www.mdpi.com/article/10.3390/jof11070503/s1, Material and Methods: Alkoxyamines synthesis; EPR experiments; Figure S1: (A) Synthesis of alkoxyamines of interest, (B) Addition of succinic group; (C) Omission of nitroxide during the prodrug synthesis; Figure S2: High-resolution mass spectrometry chromatograms; Figure S3: Kinetics of TEMPO release (concentration over time) for Va at 132 °C; Figure S4: Enzymatic activation followed by homolysis of the alkoxyamine; Figure S5: Alkoxyamine Va alters the epithelial monolayer integrity; Table S1: EPR kinetic figures of homolysis rate constants (*k*_d_), activation energies (*E*_a_) and half-life times (t_1/2_); Equation S1: Rate constant determination, Equation S2: Arrhenius law.
