# Supplementary material for: Pathogen Enzyme-Mediated Alkoxyamine Homolysis as a Killing Mechanism of Aspergillus fumigatus
Source: J Fungi (Basel). 2025 Jul 4;11(7):503. doi: 10.3390/jof11070503 (PMC12299905; doi:10.3390/jof11070503)
Supplement: Supplementary file 1 [file jof-11-00503-s001.zip › jof-3674247-supplementary.pdf]

Supporting Information

Pathogen enzyme-mediated alkoxyamine homolysis as a killing mechanism of *Aspergillus fumigatus*

Marion Filliâtre[1], Pierre Voisin[2], Seda Seren[2], Ines Kelkoul [2], Olivier Glehen[3], Philippe Mellet[2,*], Sophie Thétiot-Laurent[1], Jean Menotti[4], Sylvain R. A. Marque[1,*], Gérard Audran[1,*], Abderrazzak Bentaher[5,*]

[1] Aix-Marseille University, CNRS, ICR-UMR 7273, Marseille 13007, France

[2] Bordeaux University, CNRS, MRBS-UMR 5536, Bordeaux 33076, France

[3] UCBL1-CICLY, Department of Digestive Surgery, Lyon University Hospital, Lyon 69000, France

[4] UCBL1-CICLY, Department of Parasitology and Mycology, la Croix-Rousse Department of Parasitology and Mycology, La Croix-Rousse Hospital, Lyon 69004, France

[5] UCBL1-CICLY-UR3738 and INSERM, Pierre-Bénite 69495, France

Alkoxyamines synthesis

Solvents and reactants for the preparation of alkoxyamines were used as received. Routine monitoring reaction was performed using silica gel 60 F254 TLC plates; spots were visualized upon exposure to UV light and a phosphomolybdic acid solution in EtOH, followed by heating. Purifications were performed on Reveleris X2 Flash System BUCHI switzerland. Cartouches flash Reveleris and GraceResolv: silica 40 µm. ^{1}H and ^{13}C NMR spectra were recorded in $CDCl_3$, CD_3OD, DMSO-d_6 on a 300 or 400 MHz spectrometer. Chemical shifts (δ) in ppm were reported using residual nondeuterated solvents as internal references for ^{1}H, ^{19}F and ^{13}C NMR spectra. High-resolution mass spectra (HRMS) were obtained on a SYNAPT G2 HDMS (Waters) spectrometer equipped with a pneumatically assisted atmospheric pressure ionization source. Positive mode electrospray ionization was used on samples: electrospray voltage: 2800 V; opening voltage: 20 V; nebulizer gas pressure (nitrogen): 800 L/h. The parent ion $[M + H]^+$ is quoted. Infrared spectra were obtained from Cary 630 FTIR Spectrometer from Agilent Technologies. Melting points have been observed with Stuart SMP10 melting point apparatus. Homolysis constants were measured by EPR on a EMX Bruker spectrometer, with O_2 as alkyl radical scavenger. Purity of final compounds was >95% as assessed by NMR spectra for all compounds and by HPLC for compounds **Va**, **VIa**, **Vb** and **VIb**.

Synthesis of alkoxyamines of interest

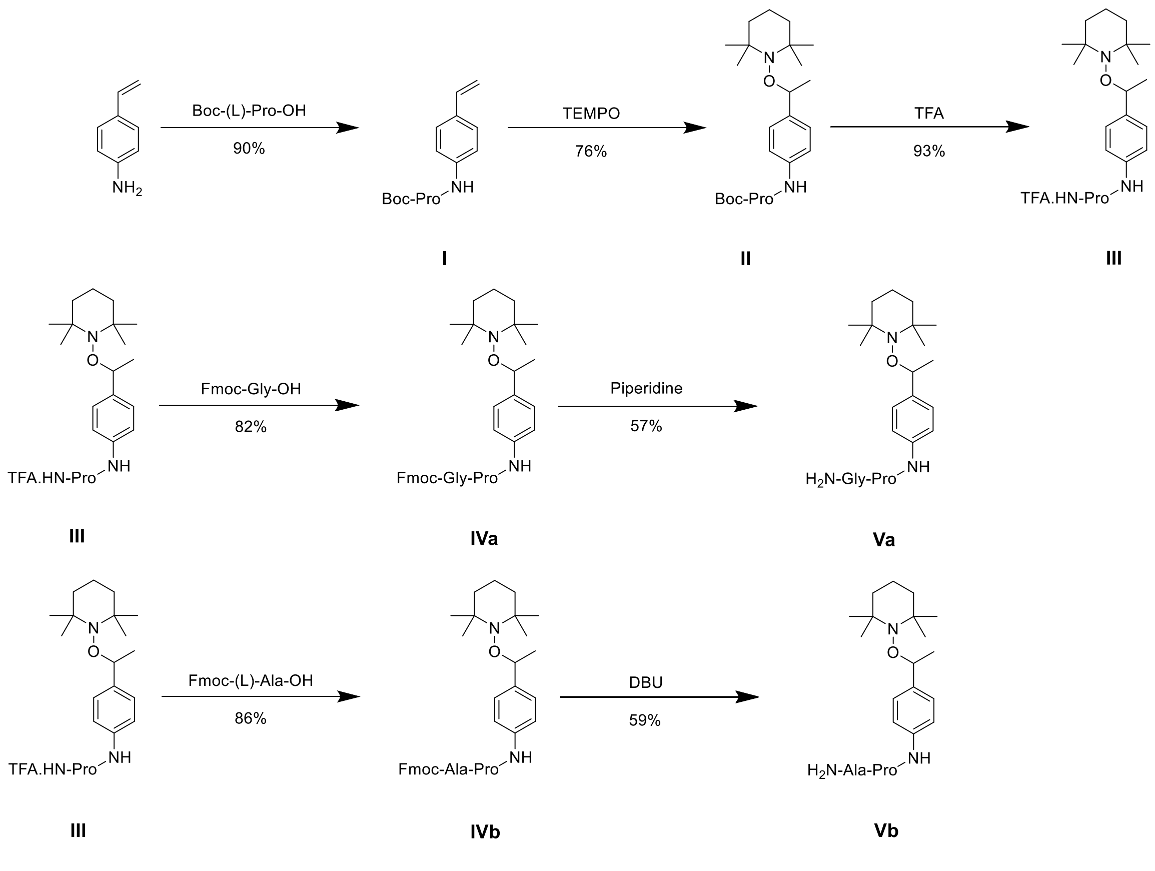

I: tert-butyl (S)-2-((4-vinylphenyl)carbamoyl)pyrrolidine-1-carboxylate.

To a solution of Boc-(L)-Pro-OH (5.96 g, 27.7 mmol, 1.1 eq.) in dry THF (100 mL) was added triethylamine (4.42 mL, 32.7 mmol, 1.3 eq.) dropwise at -15 °C. Five minutes later, ethyl chloroformate (3.11 mL, 32.7 mmol, 1.3 eq.) was added dropwise and the mixture was stirred at this temperature for an hour. Then 4-vinylaniline (3.00 g, 25.2 mmol, 1.0 eq.) dissolved in dry THF (20 mL) was added by canulation to the suspension. The mixture was stirred at -15°C for 30 minutes and then was slowly brought to rt and stirred overnight. The solvent was evaporated, and the mixture was dissolved in EtOAc (150 mL). The organic phase was washed with HCl 1M (80 mL), $NaHCO_{3(sat)}$ (80 mL), $NaCl_{(sat)}$ (80 mL), dried over $MgSO_4$, filtered, and concentrated under reduced pressure. The crude product was purified by column chromatography (silica column, PE/EtOAc gradient from 7:3 to 5:5) to afford a white powder (7.14 g, 90%). ^{1}H NMR (400 MHz, $CDCl_3$) δ (ppm) 9.51 (s, 1H, H_9), 7.48 (d, J = 8.6 Hz, 2H, H_4/H_8), 7.34 (d, J = 8.2 Hz, 2H, H_5/H_7), 6.66 (dd, J = 10.9 and 17.6 Hz, 1H, H_1), 5.66 (d, J = 17.6 Hz, 1H, H_{3a}), 5.17 (d, J = 10.9 Hz, 1H, H_{3b}), 4.52–4.26 (m, 1H, H_{12}), 3.58–3.29 (m, 2H, H_{15}), 2.62–2.21 (m, 1H, H_{13}), 2.06–1.84 (m, 3H, H_{13}/H_{14}), 1.49 (s, 9H, H_{21}).

II: tert-butyl (2S)-2-((4-(1-((2,2,6,6-tetramethylpiperidin-1 yl)oxy)ethyl)phenyl)carbamoyl) pyrrolidine-1-carboxylate.

To a solution of Salen ligand (578 mg, 2.15 mmol, 0.15 eq.) in isopropyl alcohol (23 mL) was added $MnCl_2$ (271 mg, 2.15 mmol, 0.15 eq.). The mixture was stirred at rt for 30 minutes. In another flask, TEMPO (2.24 g, 14.4 mmol, 1.0 eq.) was added to a solution of **1** (5.00 g, 15.8 mmol, 1.1 eq.) in isopropyl alcohol (23 mL). This mixture was poured in the $MnCl_2$/Salen suspension. Then, sodium borohydride (2.17 g, 57.4 mmol, 4.0 eq.) was added in four portions, each at a 15-minute spacing. The mixture was then firmly stirred at rt overnight. EtOAc (100 mL) was added, and the excess of sodium borohydride was neutralized with HCl 1M addition while still under stirring. Once there was no off-gas, $NaHCO_{3(sat)}$ was added until neutralization. The organic phase was washed with $NaCl_{(sat)}$, dried over $MgSO_4$, filtered and concentrated under reduced pressure. The crude product was purified by column chromatography (40 g silica column, PE/EtOAc gradient from 9:1 to 6:4) to afford white powder (5.70 g, 76%). ^{1}H NMR (400 MHz, DMSO-d6) δ (ppm) 9.89 (s, 1H, H_9), 7.54 (d, J = 8.4 Hz, 2H, H_4/H_8), 7.23 (d, J = 8.1 Hz, 2H, H_5/H_7), 4.69 (q, J = 6.5 Hz, 1H, H_1), 4.26–4.16 (m, 1H, H_{12}), 3.45–3.32 (m, 2H, H_{15}), 2.24–1.76 (m, 4H, H_{13}/H_{14}), 1.48–1.26 (m, 21H, H_3/$H_{21–23}$/H_{25}/H_{32}), 1.11 (s, 3H, H_{27}), 0.96 (s, 3H, H_{26}), 0.60–0.56 (m, 3H, H_{28}). ^{13}C NMR (101 MHz, DMSO-d6) **)** δ (ppm) 171.3 (C=O_{dia1}), 170.9 (C=O_{dia2}), 153.6 (C=O_{dia2}), 153.1 (C=O_{dia1}), 139.9 (C, C_{6dia2}), 139.8 (C, C_{6dia1}), 137.9 (C, C_2), 126.9 (2 × CH, $(C_4/C_8)_{dia1}$), 126.7 (2 × CH, $(C_4/C_8)_{dia2}$), 118.9 (2 × CH, $(C_5/C_7)_{dia1}$), 118.8 (2 × CH, $(C_5/C_7)_{dia2}$), 82.0 (CH, C_{1dia2}), 81.9 (CH, C_{1dia1}), 78.6 (C, C_{31dia2}), 78.4 (C, C_{31dia1}), 60.3 (CH, C_{12dia1}), 59.9 (CH, C_{12dia2}), 59.2 (C, $C_{20 \text{ or } 24}$), 58.9 (C, $C_{20 \text{ or } 24}$), 46.7 (CH_2, C_{15dia2}), 46.5 (CH_2, C_{15dia1}), 39.7 (2 × CH_2, C_{21}/C_{23}), 30.9 (CH_2, $C_{(13 \text{ or } 14)dia1}$), 30.1 ($CH_2$, $C_{(13 \text{ or } 14)dia2}$), 28.1 (3 × CH_3, C_{32dia2}), 27.9 (3 × CH_3, C_{32dia1}), 23.9 (CH_2, $C_{(13 \text{ or } 14)dia2}$), 23.3 ($CH_2$, $C_{(13 \text{ or } 14)dia1}$), 23.0 (4 × CH_3, $C_{(25–28)dia2}$), 22.7 (4

× CH_3, $C_{(25–28)dia1}$), 20.0 (CH_3, C_3), 16.7 (CH_2, C_{22}). HRMS (ESI) calc. for $C_{27}H_{44}N_3O_4^+$: 474.3326 [M + H]$^+$; found: 474.3325 Da. R_F = 0.37 (EtOAc/PE 4:6). Mp = 148 °C. IR υ (cm^{-1}) 2950, 1700, 1670.

III: (2S)-2-((4-(1-((2,2,6,6-tetramethylpiperidin-1-yl)oxy)ethyl)phenyl)carbamoyl)pyrrolidin-1-ium.

To a solution of **2** (3.02 g, 6.37 mmol, 1.0 eq.) in DCM (30 mL) was added trifluoroacetic acid (4.87 mL, 63.7 mmol, 10.0 eq.) dropwise at 0 °C. The mixture was brought to rt and stirred for 23 h. DCM and trifluoroacetic acid were evaporated under reduced pressure. The crude product was purified by column chromatography (silica column, DCM/MeOH 9:1) to afford an orange oil product (955 mg, 93%). ^{1}H NMR (400 MHz, $CDCl_3$) δ (ppm) 10.69 (s, 2H, H_{16}), 7.55 (d, *J* = 7.6 Hz, 2H, H_4/H_8), 7.31 (d, *J* = 7.8 Hz, 2H, H_5/H_7), 5.62 (d, *J* = 6.2 Hz, 1H, H_9), 4.63 (br s, 1H, H_1), 3.45–3.39 (m, partially overlapped, 1H, H_{12}), 2.47 (s, 1H, H_{15a}), 2.14–1.99 (m, 5H, H_{13}/H_{14}/H_{15b}), 1.67–1.53 (m, 9H, H_3/$H_{20–22}$), 1.50 (s, 3H, H_{24}), 1.39 (s, 3H, H_{26}), 1.18 (s, 3H, H_{25}), 0.84 (s, 3H, H_{27}). ^{19}F NMR (376 MHz, $CDCl_3$) δ (ppm) 75.55. ^{13}C NMR (101 MHz, $CDCl_3$) δ (ppm) 167.1 (C=O), 138.2 (C, C_6), 137.4 (C, C_2), 127.6 (2 × CH, C_4/C_8), 120.4 (2 × CH, C_5/C_7), 85.5 (CH, C_1), 69.5 (C, $C_{19\text{ or }23dia1}$), 68.8 (C, $C_{19\text{ or }23dia2}$), 60.5 (CH, C_{12}), 46.7 (CH_2, C_{15}), 37.3 (CH_2, $C_{13\text{ or }14}$), 30.1 (CH_2, $C_{13\text{ or }14}$), 29.4 (C, $C_{19\text{ or }23dia1}$), 29.1 (C, $C_{19\text{ or }23dia2}$), 24.5 (CH_2, $C_{20\text{ or }22}$), 24.4 (CH_2, $C_{20\text{ or }22}$), 23.2 (CH_3, C_3), 20.9 (2 × CH_3, C_{24}/C_{26}), 20.7 (2 × CH_3, C_{25}/C_{27}), 15.8 (CH_2, C_{21}). HRMS (ESI) calc. for $C_{22}H_{36}N_3O_2^+$: 374.2802 [M + H]$^+$; found: 374.2803 Da. R_F = 0.40 (DCM/MeOH 9:1). Mp = 95 °C. IR υ (cm^{-1}) 3080, 2950, 1680.

IVa: (9H-fluoren-9-yl)methyl(2-oxo-2-((2S)-2-((4-(1-((2,2,6,6-tetramethylpiperidin-1-l)oxy)ethyl)phenyl)carbamoyl)pyrrolidin-1-yl)ethyl)carbamate and

IVb: (9H-fluoren-9-yl)methyl ((2S)-1-oxo-1-((2S)-2-((4-(1-((2,2,6,6-tetramethylpiperidin-1-yl)oxy)ethyl)phenyl)carbamoyl)pyrrolidin-1-yl)propan-2-yl)carbamate.

To a solution of **III** (1.92 g, 3.94 mmol, 1.0 eq. for **IVa** and 2.50 g, 5.13 mmol, 1.0 eq. for **IVb**) in dry DCM (40 mL) was added DIPEA (1.0 eq.) dropwise at 0 °C. Ten minutes later, Fmoc-(L)-Gly-OH (1.25 g, 4.33 mmol, 1.1 eq.) or Fmoc-Ala-OH (1.60 g, 5.13 mmol, 1.0 eq.) and HOBt (1.1 eq.) were added at 0 °C. The mixture was stirred for 15 minutes and then EDCI (1.1 eq.) was added. The mixture was stirred at rt for 18 h. The organic phase was washed with HCl 1M (20 mL), $NaHCO_{3(sat)}$ (20 mL), water (20 mL), brine (20 mL), dried over $MgSO_4$, filtered, and concentrated under reduced pressure. The crude product was purified by column chromatography (silica column, PE/EtOAc gradient from 4:6 to 2:8) to afford white powders **IVa** (2.12 g, 82%) and **IVb** (2.94 g, 86%).

IVa: ^{1}H NMR (400 MHz, MeOD) δ (ppm) 7.74-7.18 (m, 12H, H_4/H_5/H_7/H_8/$H_{41–48}$), 4.71 (q, partially overlapped, *J* = 6.1 Hz, 1H, H_1), 4.52 (br s, 1H, H_{12}), 4.28 (d, *J* = 6.7 Hz, 2H, H_{35}), 4.16–4.13 (t, *J* = 6.4 Hz, 1H, H_{36}), 3.97 (dd, *J* = 17.0 and 15.4 Hz, 2H, H_{30}), 3.83–3.45 (m, 2H, H_{15}), 2.20–1.85 (m, 4H, H_{13}/H_{14}), 1.40 (d, *J* = 6.5 Hz, overlapped, 3H, H_3), 1.50–0.59 (m, 18H, $H_{20–22}$/$H_{24–27}$). ^{13}C NMR (101 MHz, MeOD δ (ppm) 172.5 (C=O), 170.3 (C=O), 158.9 (C=O), 145.2 (2 × C, C_{arom}), 142.7 (C, C_{arom}), 142.4 (2 × C, C_{arom}), 138.3 (C, C_{arom}), 128.7–120.9 (12 × CH,

$C_4/C_5/C_7/C_8/C_{41–48}$), 83.9 (CH, C_1), 68.1 (CH_2, C_{35}), 62.4 (CH, C_{12}), 60.8 (C, $C_{19 \text{ or } 23}$), 48.4 (CH, C_{36}), 47.8 (CH_2, $C_{15 \text{ or } 30}$), 44.2 (CH_2, $C_{15 \text{ or } 30}$), 41.3 (2 × CH_2, C_{20}/C_{22}), 34.7 (C, $C_{19 \text{ or } 23}$), 30.6 (CH_2, $C_{13 \text{ or } 14}$), 25.8 (CH_2, $C_{13 \text{ or } 14}$), 23.7 (CH_3, C_3), 20.9 (4 × CH_3, $C_{24–27}$), 18.1 (CH_2, C_{21}). HRMS (ESI) calc. for $C_{39}H_{49}N_4O_5^+$: 653.3697 [M + H]$^+$; found: 653.3695 Da. R_F = 0.31 (EtOAc/PE 7:3). Mp = 108 °C. IR υ (cm^{-1}) 3290, 2910, 1695, 1650, 1605.

IVb: ^{1}H NMR (400 MHz, $CDCl_3$) *δ* (ppm) 9.16 (s, 1H, NH), 7.77–7.22 (m, 12H, $H_{4/H5}/H_7/H_8/H_{42–49}$), 5.83 (d, *J* = 7.9 Hz, 1H, NH), 4.79–4.71 (m, 2H, H_1/H_{29}), 4.59 (t, *J* = 7.3 Hz, 1H, $H_{12 \text{ or } 37}$), 4.36 (dd, *J* = 7.8 Hz, 2H, H_{36}), 4.20 (t, *J* = 7.1 Hz, 1H, $H_{12 \text{ or } 37}$), 3.73–3.56 (m, 2H, H_{15}), 2.50–2.14 (m, 2H, $H_{13 \text{ or } 14}$), 2.05–1.86 (m, 2H, $H_{13 \text{ or } 14}$), 1.43 (d, J = 6.7 Hz, overlapped, 3H, $H_{3 \text{ or } 32}$), 1.41 (d, *J* = 6.9 Hz, overlapped, 3H, $H_{3 \text{ or } 32}$), 1.60–1.35 (m, 6H, $H_{20–22}$), 1.28 (s, 3H, H_{24}), 1.16 (s, 3H, H_{26}), 1.02 (s, 3H, H_{25}), 0.67 (s, 3H, H_{27}). ^{13}C NMR (101 MHz, $CDCl_3$) *δ* (ppm) 173.5 (C=O), 168.7 (C=O), 155.9 (C=O), 143.9 (2 x C, C_{arom}), 141.8 (C, C_6), 141.4 (2 × C, C_{arom}), 136.9 (C, C_2), 127.8-119.5 (12 × CH, $C_4/C_5/C_7/C_8/C_{42–49}$), 82.7 (CH, C_1), 67.1 (CH_2, C_{36}), 60.9 (CH, C_{12}), 59.7 (2 × C, C_{19}/C_{23}), 49.2 (CH, $C_{(29 \text{ or } 37)dia2}$), 48.5 (CH, $C_{(29 \text{ or } 37)dia1}$), 47.5 ($CH_2$, C_{15}), 47.2 (CH, $C_{29 \text{ or } 37}$), 40.4 (2 × CH_2, C_{20}/C_{22}), 34.5 (2 × CH_3, C_{24}/C_{26}), 26.9 (CH_2, $C_{(13 \text{ or } 14)dia1}$), 25.7 ($CH_2$, $C_{(13 \text{ or } 14)dia2}$), 25.2 ($CH_2$, $C_{(13 \text{ or } 14)dia1}$), 25.0 ($CH_2$, $C_{(13 \text{ or } 14)dia2}$), 23.5 ($CH_3$, $C_{3 \text{ or } 32}$), 20.4 (2 × CH_3, C_{25}/C_{27}), 18.6 (CH_3, $C_{3 \text{ or } 32}$), 17.3 (CH_2, C_{21}). HRMS (ESI) calc. for $C_{40}H_{51}N_4O_5^+$: 667.3854 [M + H]$^+$; found: 667.3853 Da. R_F = 0.55 (EtOAc/PE 8:2). Mp = 110 °C. IR υ (cm^{-1}) 3280, 2910, 1690, 1610.

Va: (2S)-1-glycyl-N-(4-(1-((2,2,6,6-tetramethylpiperidin-1-yl)oxy)ethyl)phenyl)pyrrolidine-2-carboxamide.

IVa (724 mg, 1.11 mmol) was dissolved in piperidine (3.50 mL). The mixture was stirred at rt for 7 h. The solvent was evaporated. The crude product was purified by flash chromatography (silica column, DCM/MeOH gradient from 100:0 to 9:1) to afford a white solid (272 mg, 57%). ^{1}H NMR (400 MHz, MeOD) *δ* (ppm) 7.64 (d, *J* = 8.7 Hz, 2H, H_4/H_8), 7.42 (d, *J* = 8.6 Hz, 2H, H_5/H_7), 5.56 (q, *J* = 6.4 Hz, 1H, H_1), 4.60 (dd, *J* = 3.8 and 8.4 Hz, 1H, H_{12}), 3.93 (s, 2H, H_{30}), 3.69–3.54 (m, 2H, H_{15}), 2.57–1.76 (m, 10H, $H_{13}/H_{14}/H_{20–22}$), 1.71 (d, *J* = 6.6 Hz, 3H, H_3), 1.68 (s, 3H, H_{24}), 1.52 (s, 3H, H_{26}), 1.32 (s, 3H, H_{25}), 0.95 (s, 3H, H_{27}). ^{13}C NMR (101 MHz, $CDCl_3$) δ (ppm) *δ* (ppm) 172.9 (C=O), 169.0 (C=O), 141.8 (C, C_6), 137.0 (C, C_2), 127.9 (CH, C_4) , 127.2 (CH, C_8), 119.6 (CH, C_5), 114.8 (CH, C_7), 82.8 (CH, C_1), 61.1 (CH, C_{12}), 59.8 (2 × C, C_{19}/C_{23}), 46.5 (CH_2, C_{15}), 44.0 (CH_2, C_{30}), 40.5 (2 × CH_2, C_{20}/C_{22}), 34.5 (CH_3, C_3), 27.2 (CH_2, $C_{13 \text{ or } 14}$), 25.1 (CH_2, $C_{13 \text{ or } 14}$), 23.6 (2 × CH_3, C_{24}/C_{26}), 20.4 (2 × CH_3, C_{25}/C_{27}), 17.3 (CH_2, C_{21}). HRMS (ESI) calc. for $C_{23}H_{39}N_4O_3^+$: 431.3017 [M + H]$^+$; found: 431.3014 Da. R_F = 0.15 (DCM/MeOH 98:2). Mp = 104 °C. IR υ (cm^{-1}) 2970, 2910, 2880, 1660, 1605.

Vb: (2S)-1-(L-alanyl)-N-(4-(1-((2,2,6,6-tetramethylpiperidin-1 yl)oxy)ethyl)phenyl)pyrrolidine-2-carboxamide.

To a solution of **IVb** (2.61 g, 3.91 mmol, 1.0 eq.) in dry DCM (40 mL) was added DBU (0.64 mL, 4.30 mmol, 1.1 eq.) dropwise at 0 °C. The mixture was stirred at 0 °C for 2 h. The solution was concentrated under reduced pressure. The crude product was purified by flash chromatography (silica column, DCM/MeOH gradient from 100:0 to 80:20) to afford a white solid (1.02 g, 59%). ^{1}H NMR (400 MHz, MeOD) *δ* (ppm) 7.64 (d, *J* = 8.7 Hz, 2H, H_4/H_8), 7.42 (d,

J = 8.6 Hz, 2H, H_5/H_7), 5.54 (q, *J* = 6.5 Hz, 1H, H_1), 4.61 (dd, *J* = 8.4 and 5.4 Hz, 1H, H_{12}), 4.29 (q, *J* = 6.9 Hz, 1H, H_{29}), 3.78–3.63 (m, 2H, H_{15}), 2.39–1.77 (m, 10H, $H_{13}/H_{14}/H_{20-22}$), 1.72 (d, *J* = 6.5 Hz, 3H, $H_{3\ or\ 32}$), 1.68 (s, 3H, H_{24}), 1.54 (d, *J* = 7.0 Hz, 3H, $H_{3\ or\ 32}$), 1.52 (s, 3H, H_{26}), 1.32 (s, 3H, H_{25}), 0.95 (s, 3H, H_{27}). ^{13}C NMR (101 MHz, MeOD) δ (ppm) 172.3 (C=O), 169.6 (C=O), 140.4 (C, C_6), 137.5 (C, C_2), 128.6 (2 × CH, C_4/C_8), 121.2 (2 x CH, C_5/C_7), 88.1 (CH, C_1), 72.3 (C, $C_{19\ or\ 23}$), 71.5 (C, $C_{19\ or\ 23}$), 62.3 (CH, C_{12}), 49.2 (CH, C_{29}), 48.5 (CH_2, C_{15}), 38.4 (CH_2, C_{20}), 38.3 (CH_2, C_{22}), 30.7 (CH_2, $C_{13\ or\ 14}$), 29.9 (CH_3, C_{24}), 29.5 (CH_3, C_{26}), 26.1 (CH_2, $C_{13\ or\ 14}$), 23.5 (CH_3, $C_{3\ or\ 32}$), 20.8 (CH_3, C_{25}), 20.7 (CH_3, C_{27}), 16.4 (CH_2, C_{21}), 16.1 (CH_3, $C_{3\ or\ 32}$). HRMS (ESI) calc. for $C_{25}H_{41}N_4O_3^+$: 445.3173 [M + H]$^+$; found: 445.3169 Da. R_F = 0.42 (DCM/MeOH 9:1). Mp = 75 °C. IR υ (cm^{-1}) 3280, 2970, 2910, 1680, 1600.

Controls of alkoxyamines of interest

Addition of succinic group during the synthesis

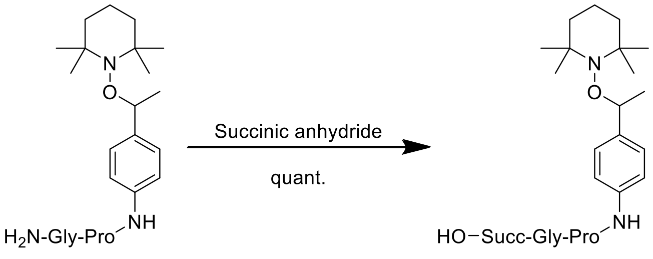

Va **VIa**

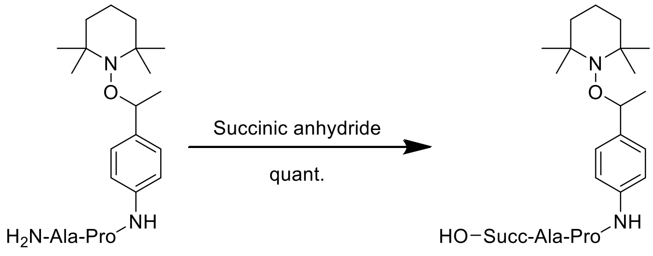

Vb **VIb**

VIa: 4-oxo-4-((2-oxo-2-((2S)-2-((4-(1-((2,2,6,6-tetramethylpiperidin-1-yl)oxy)ethyl)phenyl) carbamoyl)pyrrolidin-1-yl)ethyl)amino)butanoic acid

and

VIb: 4-oxo-4-(((2S)-1-oxo-1-((2S)-2-((4-(1-((2,2,6,6-tetramethylpiperidin-1-yl)oxy)ethyl)phenyl)carbamoyl)pyrrolidin-1-yl)propan-2-yl)amino)butanoic acid.

To a solution of **Va** (99.1 mg, 0.23 mmol, 1.0 eq.) or **Vb** (122.3 mg, 0.27 mmol, 1.0 eq.) in DCM (2 mL) was added succinic anhydride (1.1 eq.). The solution was stirred under air for one hour and then concentrated under reduced pressure. The crude product is pure white powders **VIa** (122.0 mg, quant.) or **VIb** (149.7 mg, quant.).

VIa: ^1H NMR (400 MHz, $CDCl_3$) δ (ppm) 9.24 (s, 1H, NH), 7.51 (d, J = 8.0 Hz, 2H, H_4/H_8), 7.38 (s, 1H, NH), 7.24 (d, J = 8.3 Hz, 2H, H_5/H_7), 4.92 (s, 1H, H_1), 4.60–4.59 (m, 1H, H_{12}), 4.12–4.09 (m, 1H, H_{30a}), 3.94–3.91 (m, 1H, H_{30b}), 3.58 (br m, 1H, H_{15a}), 3.42–3.39 (m, 1H, H_{15b}), 2.56–2.48 (m, 4H, H_{34}/H_{35}), 2.19–1.92 (m, 4H, H_{13}/H_{14}), 1.62–1.51 (m, 4H, H_{20}/H_{22}), 1.45 (d, J = 6.5 Hz, 3H, H_3), 1.35 (s, 5H, H_{21}/H_{24}), 1.17 (s, 3H, H_{26}), 1.02 (s, 3H, H_{25}), 0.70 (s, 3H, H_{27}). ^{13}C NMR (101 MHz, $CDCl_3$) δ (ppm) 175.9 (C=O), 173.4 (C=O), 170.0 (C=O), 169.1 (C=O), 141.0 (C, C_6), 137.2 (C, C_2), 127.3 (2 × CH, C_4/C_8), 119.9 (2 x CH, C_5/C_7), 83.2 (CH, C_1), 61.7 (2 x C, C_{19}/C_{23}), 61.4 (CH, C_{12}), 47.0 (CH_2, C_{15}), 42.3 (CH_2, C_{30}), 39.8 (2 × CH_2, C_{20}/C_{22}), 33.5 (2 × CH_3, C_{24}/C_{26}), 30.6 (CH_2, C_{34or35}), 29.7 (CH_2, C_{34or35}), 28.8 (CH_2, C_{13or14}), 24.9 (CH_2, C_{13or14}), 23.7 (CH_3, C_3), 20.6 (2 × CH_3, C_{25}/C_{27}), 17.0 (CH_2, C_{21}). HRMS (ESI) calc. for $C_{28}H_{42}N_4O_6Na^+$: 553.2997 $[M + Na]^+$; found: 553.2997 Da. R_F = 0.36 (DCM/MeOH 9:1). Mp = 103 °C. IR υ (cm^{-1}) 3290, 2980, 2910, 1710, 1630, 1605.

VIb: ^1H NMR (400 MHz, MeOD) δ (ppm) 7.51 (d, J = 8.5 Hz, 2H, H_4/H_8), 7.25 (d, J = 8.5 Hz, 2H, H_5/H_7), 4.75 (q, J = 6.6 Hz, 1H, $H_{1\text{ or }29}$), 4.63 (q, J = 7.0 Hz, 1H, $H_{1\text{ or }29}$), 4.53 (dd, J = 8.5 and 4.8 Hz, 1H, H_{12}), 3.92–3.65 (m, 2H, H_{15}), 2.54–2.43 (m, 4H, H_{34}/H_{35}), 2.34–1.99 (m, 4H, H_{13}/H_{14}), 1.60-1.32 (m, 9H, H_{24}/H_{20-22}), 1.45 (d, J = 6.7 Hz, 3H, $H_{3\text{ or }32}$), 1.36 (d, J = 7.0 Hz, 3H, $H_{3\text{ or }32}$), 1.18 (s, 3H, H_{26}), 1.03 (s, 3H, H_{25}), 0.62 (s, 3H, H_{27}). ^{13}C NMR (101 MHz, MeOD) δ (ppm) 179.2 (C=O), 175.1 (C=O), 173.6 (C=O), 172.7 (C=O), 142.8 (C, C_6), 138.6 (C, C_2), 128.2 (2 × CH, C_4/C_8), 120.9 (2 × CH, C_5/C_7), 84.1 (CH, C_1), 62.3 (CH, C_{12}), 61.0 (C, $C_{19\text{ or }23}$), 48.5 (CH, C_{29}), 47.7 (CH_2, C_{15}), 41.4 (2 × CH_2, C_{20}/C_{22}), 34.8 (C, $C_{19\text{ or }23}$), 33.1 (CH_2, $C_{34\text{ or }35}$), 32.8 (CH_2, $C_{34\text{ or }35}$), 30.7 (CH_2, $C_{13\text{ or }14}$), 26.1 (CH_2, $C_{13\text{ or }14}$), 23.7 (4 × CH_3, C_{24-27}), 18.2 (CH_2, C_{21}), 16.9 (2 × CH_3, C_3/C_{32}). HRMS (ESI) calc. for $C_{29}H_{45}N_4O_6^+$: 545.3334 $[M + H]^+$; found: 555.3332 Da. R_F = 0.36 (DCM/MeOH 9:1). Mp = 90 °C. IR υ (cm^{-1}) 3290, 2980, 2910, 1620, 1605, 1540.

Omission of nitroxide during the synthesis:

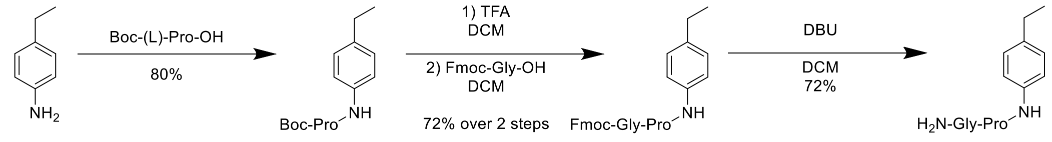

T1 **T2** **VIc**

T1: tert-butyl (S)-2-((4-ethylphenyl)carbamoyl)pyrrolidine-1-carboxylate.
To a solution of Boc-(L)-Pro-OH (6.89 g, 32.0 mmol, 1.0 eq.) in dry THF (300 mL) under inert atmosphere was added triethylamine (5.62 mL, 41.6 mmol, 1.3 eq.) dropwise at -15 °C. Five minutes later, ethyl chloroformate (3.96 mL, 41.6 mmol, 1.3 eq.) was added dropwise and the mixture was stirred at this temperature for an hour. Then, 4-ethylaniline (4.00 mL, 32.0 mmol, 1.0 eq.) was added dropwise. The mixture was stirred at -15°C for 30 minutes and then was slowly brought to rt and stirred overnight. The solvent was evaporated, and the mixture was dissolved in EtOAc (160 mL). The organic phase was washed with HCl 1M (100 mL), $NaHCO_{3(sat)}$ (100 mL), $NaCl_{(sat)}$ (100 mL), dried over $MgSO_4$, filtered, and concentrated under reduced pressure. The crude product was purified by flash chromatography (40 g silica column, PE/EtOAc gradient from 7:3 to 5:5) to afford an orange powder (8.18 g, 80%). ^1H NMR (400

MHz, $CDCl_3$) δ (ppm) 9.33 (s, 1H, H_9), 7.42 (d, J = 8.4 Hz, 2H, H_4/H_8), 7.14 (d, J = 8.1 Hz, 2H, H_5/H_7), 4.58–4.28 (m, 1H, H_{12}), 3.61–3.25 (m, 2H, H_{15}), 2.61 (q, J = 7.6 Hz, 2H, H_1), 2.58–2.43 (m, 1H, H_{13a}), 2.01–1.84 (m, 3H, H_{13b}/H_{14}), 1.49 (s, 9H, H_{21}), 1.21 (t, J = 7.6 Hz, 3H, H_3).

T2: (9H-fluoren-9-yl)methyl((S)-1-((S)-2-((4-ethylphenyl)carbamoyl)pyrrolidin-1-yl)-1-oxopropan-2-yl)carbamate.

To a solution of **T1** (6.00 g, 18.8 mmol, 1.0 eq.) in DCM (66 mL) was added trifluoroacetic acid (7.21 mL, 94.2 mmol, 5.0 eq.) dropwise at 0 °C. The mixture was brought to rt and stirred for 6 h. The mixture was concentrated under reduced pressure. The crude product was purified by column chromatography (silica column, DCM/MeOH 9:1) to afford an orange oil product (5.41 g, 86%). To a solution of this molecule (2.96 g, 8.90 mmol, 1.0 eq.) in dry DCM (90 mL) was added DIPEA (1.51 mL, 8.90 mmol, 1.0 eq.) dropwise at 0 °C under inert atmosphere. The mixture was stirred at this temperature for 15 minutes, then Fmoc-Ala-OH (2.77 g, 8.90 mmol, 1.0 eq.) and HOBt (1.32 g, 9.80 mmol, 1.1 eq.) were added. The mixture was stirred at for 15 minutes and then EDCI (1.87 g, 9.80 mmol, 1.1 eq.) was added. The mixture was brought to rt and stirred for 17 h. The solution was concentrated then dissolved in EtOAc (100 mL) and was washed with HCl 1M (60 mL), $NaHCO_{3(sat)}$ (60 mL), water (60 mL), brine (60 mL), dried over $MgSO_4$, filtered, and concentrated under reduced pressure. The crude product, a white powder product (3.01 g, 50%), was directly used for next step with no further purification. ^1H NMR (300 MHz, $CDCl_3$) δ (ppm) 9.14 (s, 1H, H_9), 7.78–7.10 (m, 12H, $H_4/H_5/H_7/H_8/H_{31–38}$), 5.75 (d, J = 8.0 Hz, 1H, H_{20}), 4.79 (dd, J = 8.1 and 2.4 Hz, 1H, H_{12}), 4.62–4.55 (m, 1H, H_{18}), 4.41–4.33 (m, 2H, H_{25}), 4.21 (t, J = 7.2 Hz, 1H, H_{26}), 3.73–3.56 (m, 2H, H_{15}), 2.59 (q, J = 7.6 Hz, 2H, H_1), 2.54–2.14 (m, 2H, $H_{13 \text{ or } 14}$), 2.09–1.86 (m, 2H, $H_{13 \text{ or } 14}$), 1.39 (d, J = 6.8 Hz, 3H, H_{21}), 1.20 (t, J = 7.6 Hz, 3H, H_3). ^{13}C NMR (101 MHz, $CDCl_3$) δ (ppm) 173.6 (C=O), 168.6 (C=O), 155.9 (C=O), 144.0–141.4 (4 × C, $C_{27–30}$), 140.3 (C, C_6), 135.9 (C, C_2), 128.3–119.9 (12 × CH, $C_4/C_5/C_7/C_8/C_{31–38}$), 67.2 ($CH_2$, C_{25}), 60.9 (CH, C_{12}), 48.5 (CH, $C_{18 \text{ or } 26}$), 47.6 (CH_2, C_{15}), 47.3 (CH, $C_{18 \text{ or } 26}$), 28.4 (CH_2, $C_{1 \text{ or } 13 \text{ or } 14}$), 26.7 ($CH_2$, $C_{1 \text{ or } 13 \text{ or } 14}$), 25.3 ($CH_2$, $C_{1 \text{ or } 13 \text{ or } 14}$), 18.7 ($CH_3$, C_{21}), 15.8 (CH_3, C_3). HRMS (ESI) calc. for $C_{31}H_{34}N_3O_4^+$: 512.2544 $[M + H]^+$; found: 512.2544 Da. R_F = 0.56 (EtOAc/PE 8:2). Mp = 99 °C. IR υ (cm^{-1}) 3290, 2950, 1690, 1680, 1650.

VIc: (S)-2-amino-1-((S)-2-(2-(4-ethylphenyl)acetyl)pyrrolidin-1-yl)propan-1-one.

To a solution of **T2** (2.76 g, 5.39 mmol, 1.0 eq.) in dry DCM (55 mL) was added DBU (0.89 mL, 5.93 mmol, 1.1 eq.) at 0 °C, under inert atmosphere. The mixture was stirred at this temperature for 3 h and then concentrated under reduced pressure. The crude product was purified by flash chromatography (silica column, DCM/MeOH gradient from 100:0 to 95:5) to afford a white solid (800 mg, 51%). ^1H NMR (400 MHz, $CDCl_3$) δ (ppm) 9.34 (s, 1H, H_9), 7.41 (d, J = 8.4 Hz, 2H, H_4/H_8), 7.12 (d, J = 8.3 Hz, 2H, H_5/H_7), 4.81 (d, J = 7.8 Hz, 1H, H_{20}), 3.75–3.49 (m, 4H, $H_{12}/H_{15}/H_{18}$), 2.66–2.50 (m, overlapped, 1H, $H_{13a \text{ or } 14a}$), 2.59 (q, J = 7.5 Hz, 2H, H_1), 2.25–1.77 (m, 3H, H_{13}/H_{14}), 1.29 (d, J = 6.8 Hz, 3H, H_{21}), 1.20 (t, J = 7.6 Hz, 3H, H_3). ^{13}C NMR (101 MHz, $CDCl_3$) δ (ppm) 176.7 (C=O), 169.0 (C=O), 140.1 (C, C_6), 136.0 (C, C_2), 128.6 (2 × CH, C_{4dia2}/C_{8dia2}), 128.2 (2 × CH, C_{4dia1}/C_{8dia1}), 119.9 (2 × CH, C_{5dia1}/C_{7dia1}), 115.4 (2 × CH, C_{5dia2}/C_{7dia2}), 60.8 (CH, C_{12}), 48.7 (CH, C_{18}), 47.1 (CH_2, C_{15}), 28.4 (CH_2, $C_{1 \text{ or } 13 \text{ or } 14}$), 26.8 ($CH_2$, $C_{1 \text{ or } 13 \text{ or } 14}$), 25.2

(CH$_2$, C$_{1 \text{ or } 13 \text{ or } 14}$), 21.4 (CH$_3$, C$_{21}$), 15.8 (CH$_3$, C$_3$). HRMS (ESI) calc. for $C_{16}H_{24}N_3O_2^+$: 290.1863 [M + H]$^+$; found: 290.1858 Da. R_F = 0.15 (DCM/MeOH 98:2). Mp = 104 °C. IR υ (cm^{-1}) 3280, 2960, 2890, 1700, 1620.

EPR experiments

Kinetic studies of homolysis of the NO-C bond of alkoxyamines were carried out by electron paramagnetic resonance (EPR) experiments. The evolution of nitroxide release was recorded in the presence of dioxygen, an alkyl radical scavenger which prevents recombination, shown in Scheme S1.

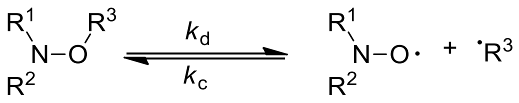

Scheme S1: Covalent bond of alkoxyamines and homolysis (k_d) and recombination (k_c) rate constants

SUPPLEMENTARY FIGURE 1

A. Synthesis of alkoxyamines of interest

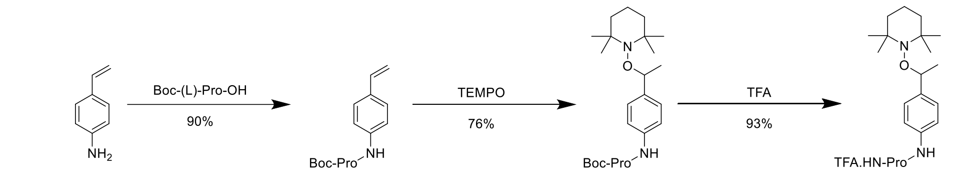

I II III

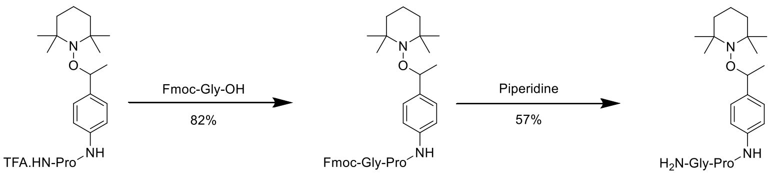

III IVa Va

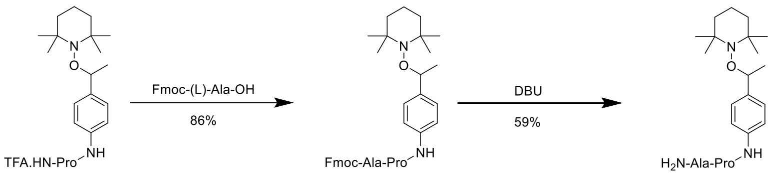

III IVb Vb

B. Addition of succinic group

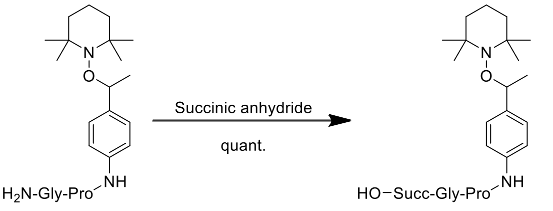

Va VIa

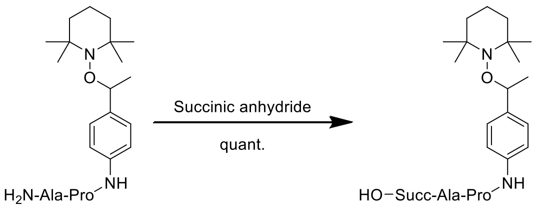

Vb VIb

C. Omission of nitroxide during the prodrug synthesis

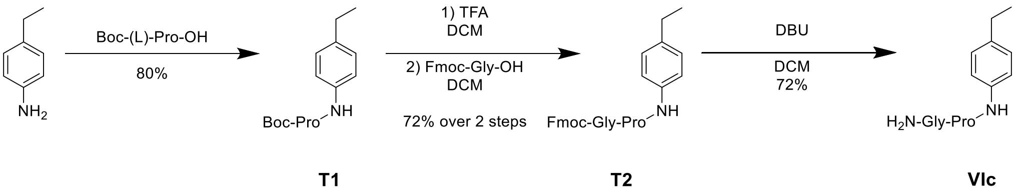

T1 T2 VIc

SUPPLEMENTARY FIGURE 2

I. ^{1}H and ^{13}C Nuclear magnetic resonance spectra

I:tert-butyl (S)-2-((4-vinylphenyl)carbamoyl)pyrrolidine-1-carboxylate

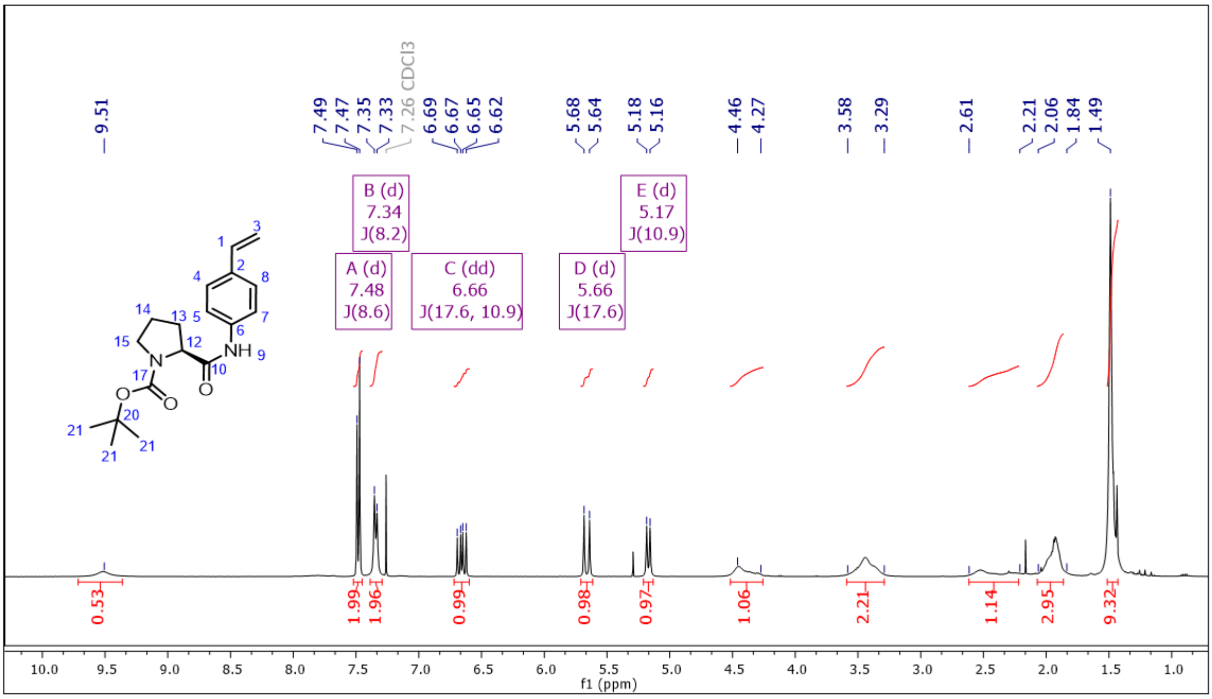

II: tert-butyl (2S)-2-((4-(1-((2,2,6,6-tetramethylpiperidin-1-yl)oxy)ethyl)phenyl)carbamoyl) pyrrolidine-1-carboxylate

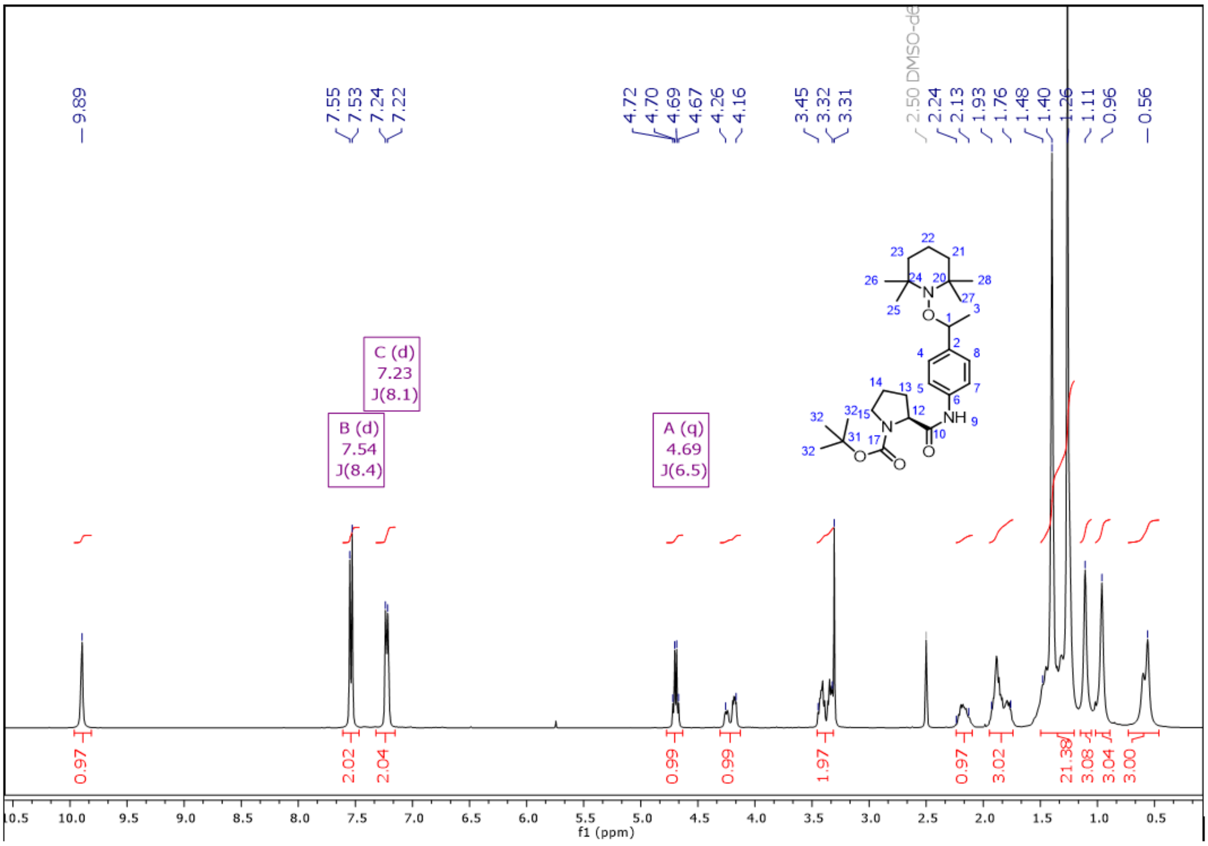

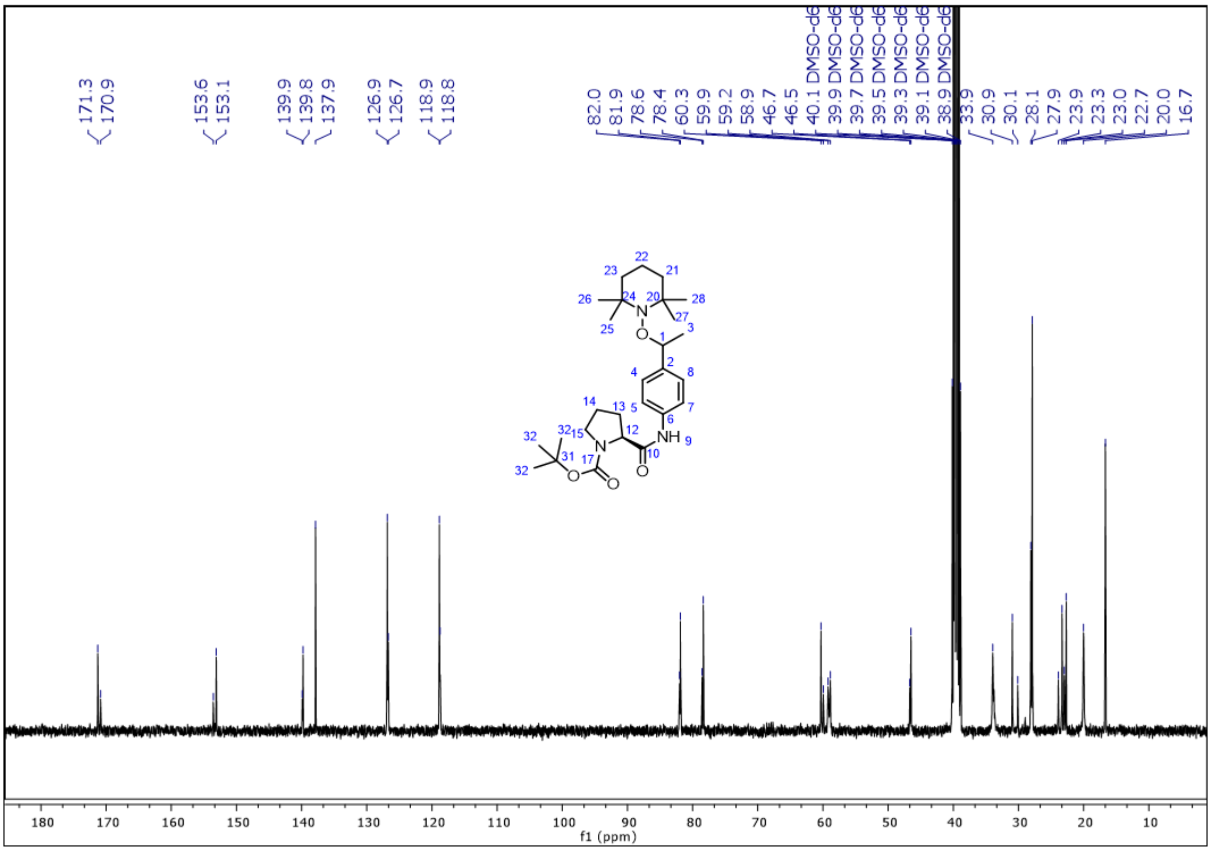

III: (2S)-2-((4-(1-((2,2,6,6-tetramethylpiperidin-1-yl)oxy)ethyl)phenyl)carbamoyl)pyrrolidin-1-ium

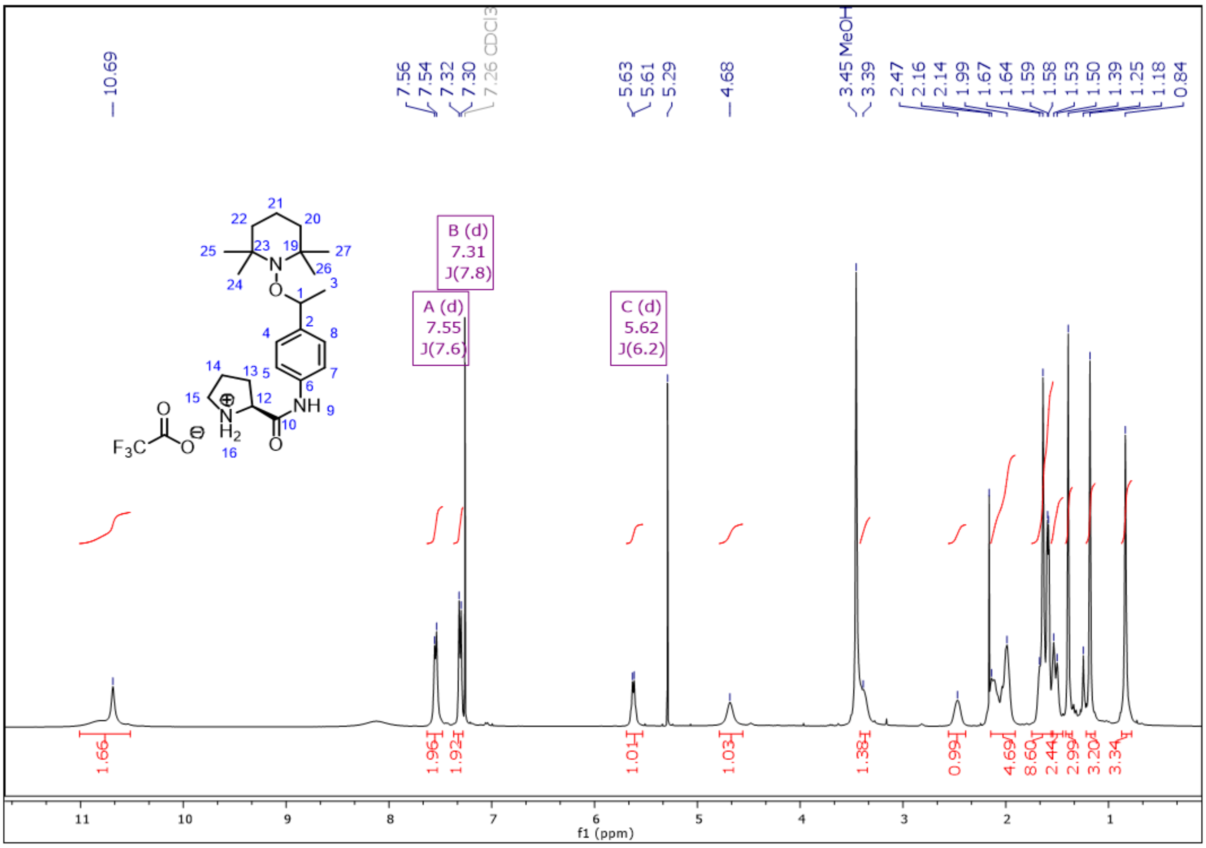

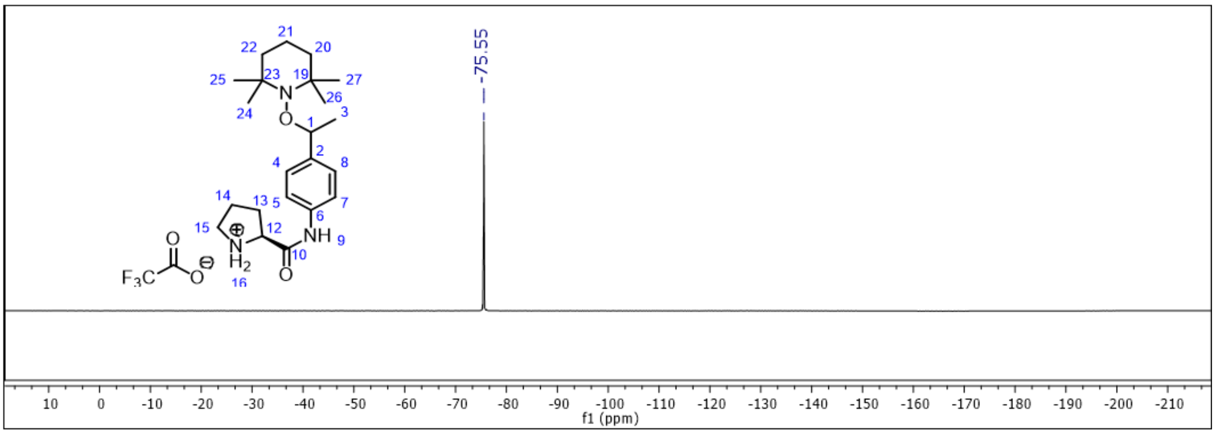

-75.55
f1 (ppm)

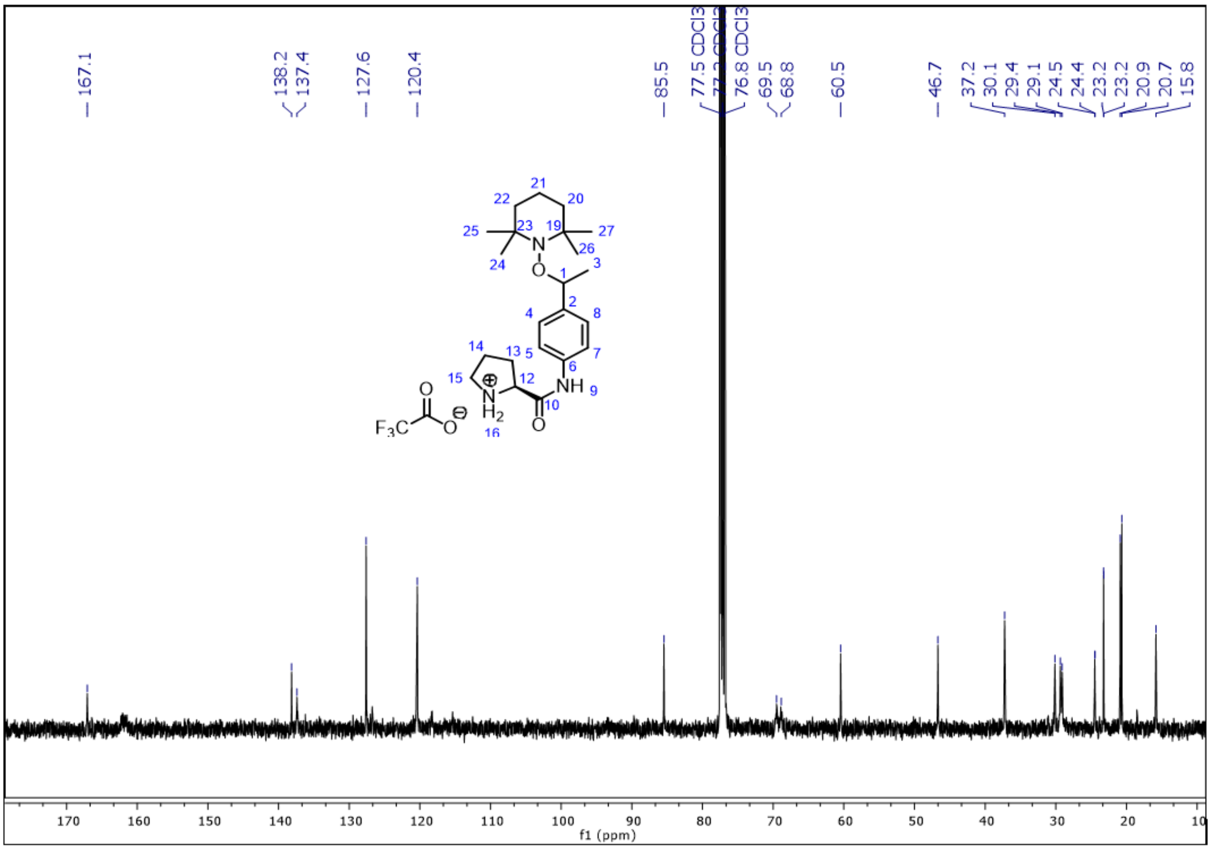

167.1
138.2
137.4
127.6
120.4
85.5
77.5 CDCl3
76.8 CDCl3
69.5
68.8
60.5
46.7
37.2
30.1
29.4
29.1
24.5
24.4
23.2
23.2
20.9
20.7
15.8
f1 (ppm)

IVa: (9H-fluoren-9-yl)methyl (2-oxo-2-((2S)-2-((4-(1-((2,2,6,6-tetramethylpiperidin-1-yl)oxy)ethyl) phenyl)carbamoyl)pyrrolidin-1-yl)ethyl)carbamate

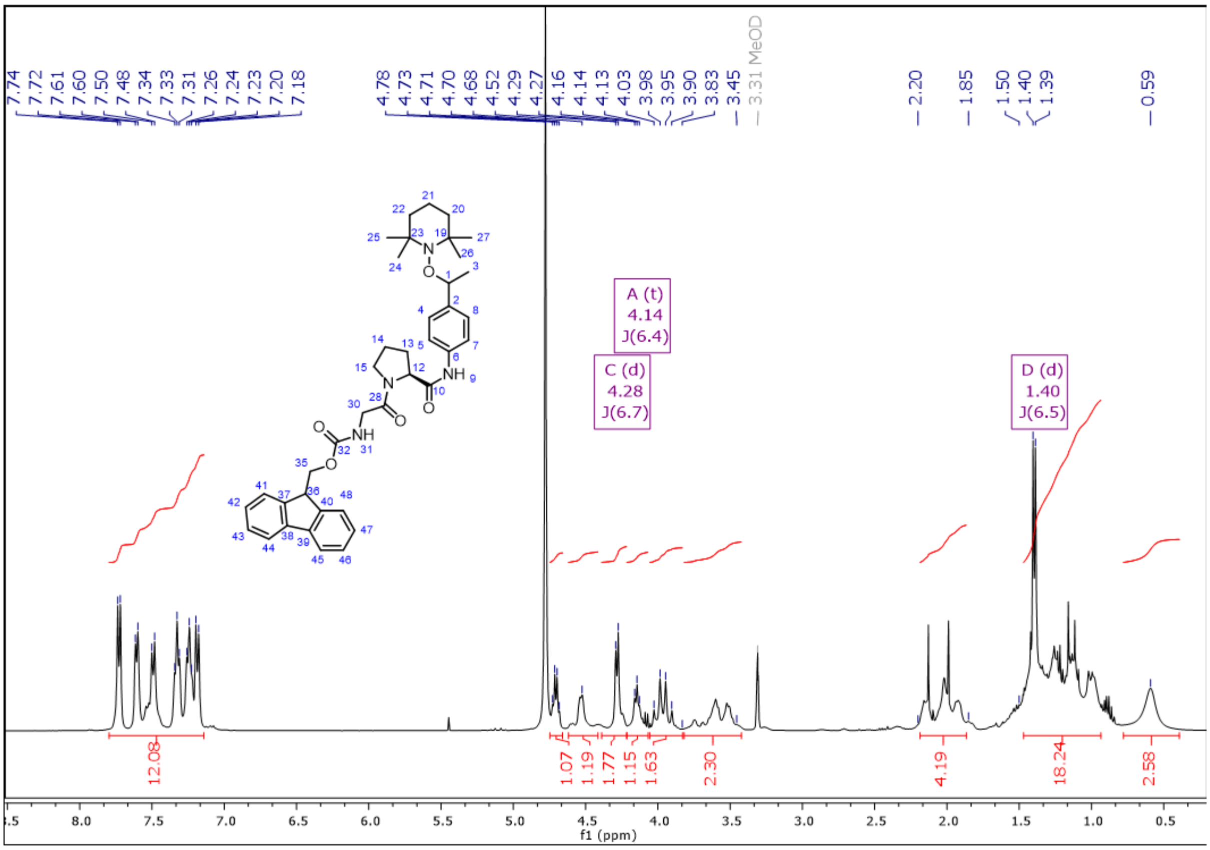

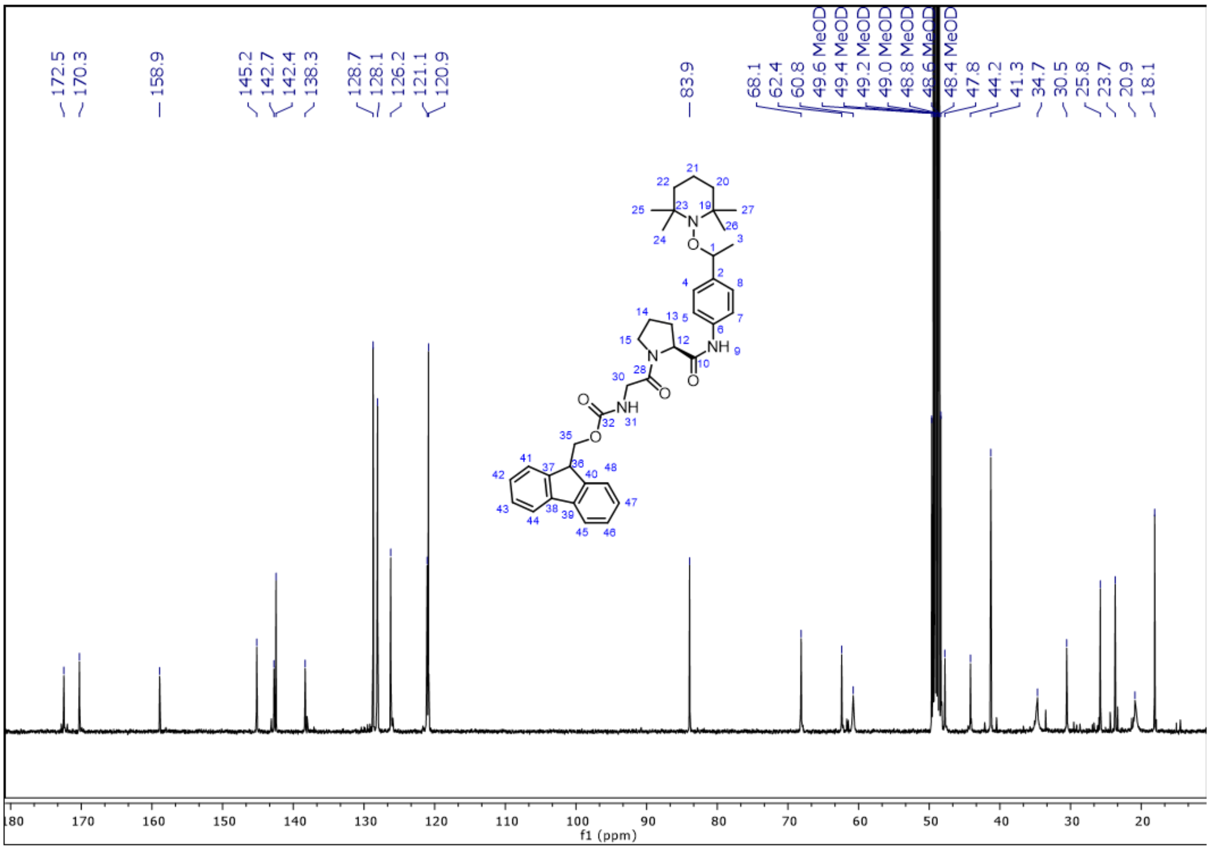

IVb: (9H-fluoren-9-yl)methyl ((2S)-1-oxo-1-((2S)-2-((4-(1-((2,2,6,6-tetramethylpiperidin-1-yl)oxy) ethyl)phenyl)carbamoyl)pyrrolidin-1-yl)propan-2-yl)carbamate

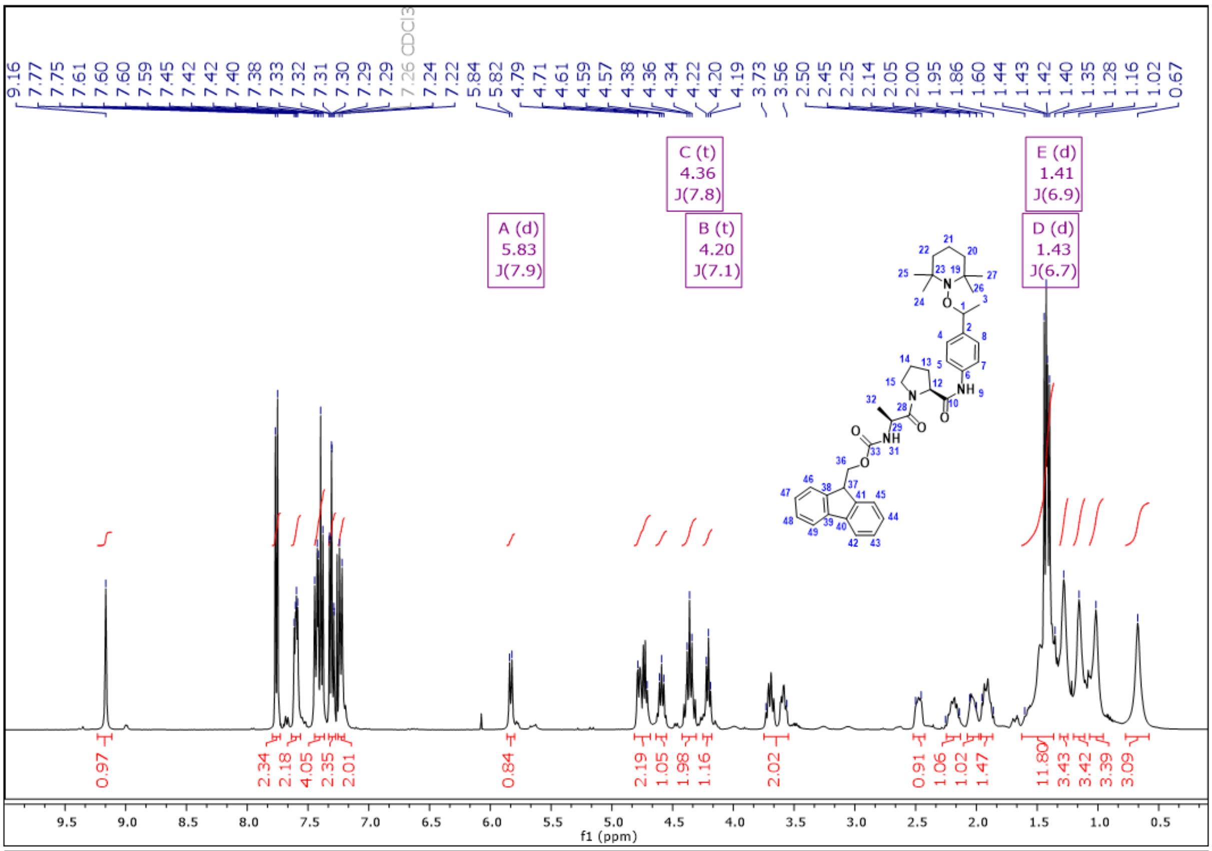

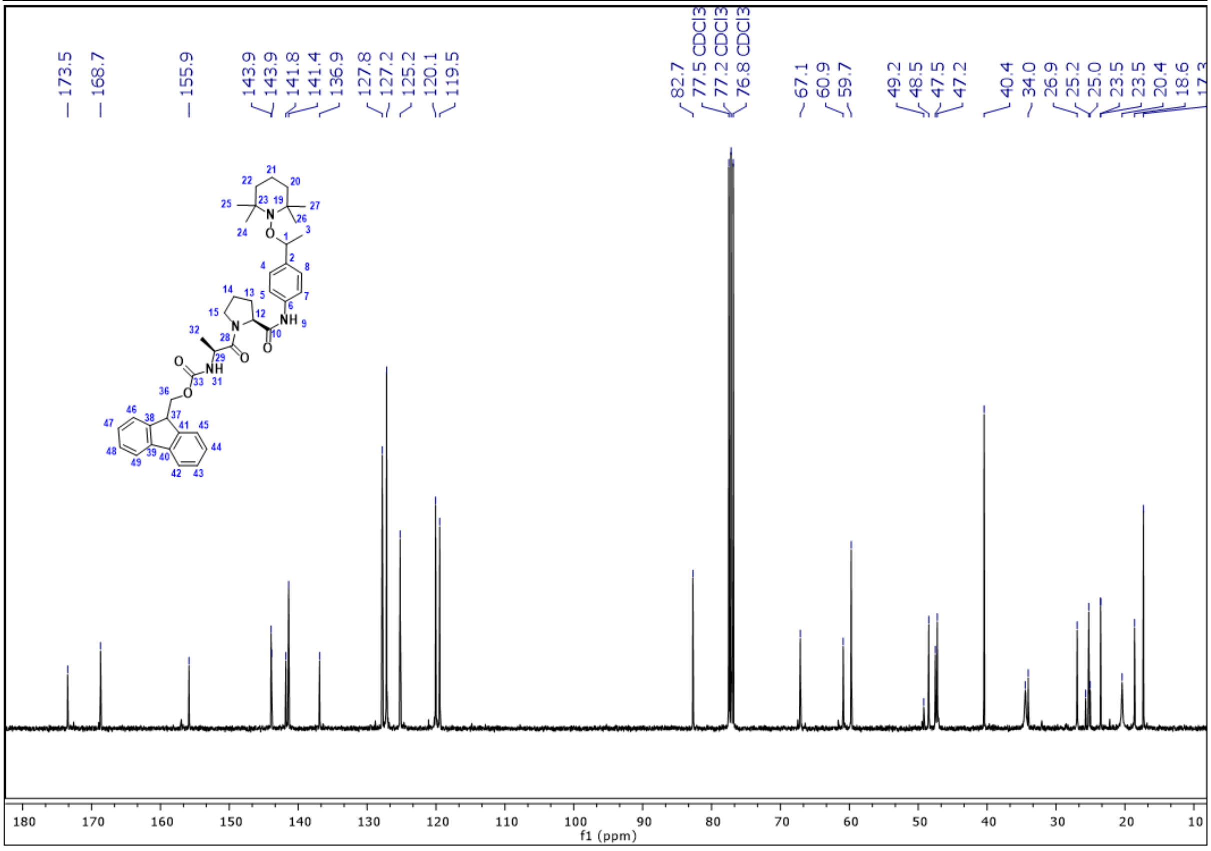

Va: (2S)-1-glycyl-N-(4-(1-((2,2,6,6-tetramethylpiperidin-1-yl)oxy)ethyl)phenyl)pyrrolidine-2-carboxamide

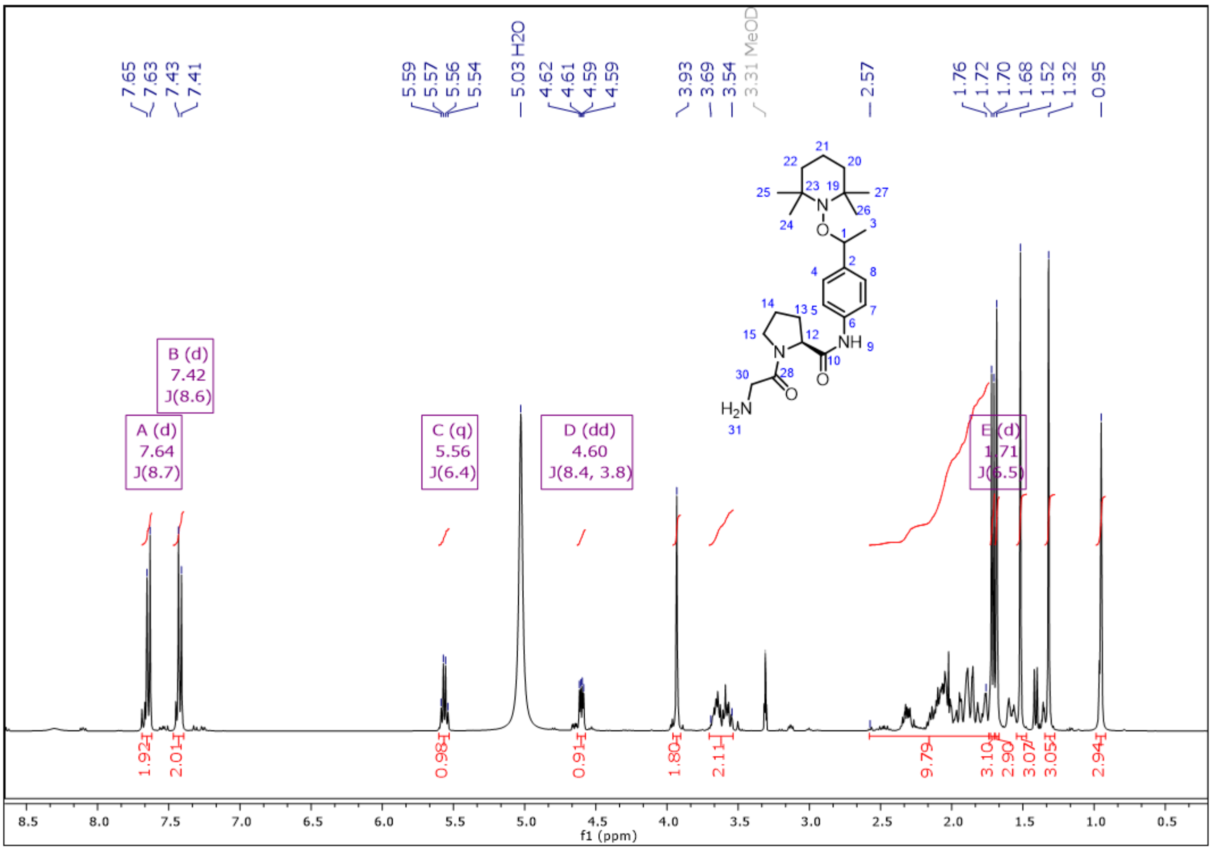

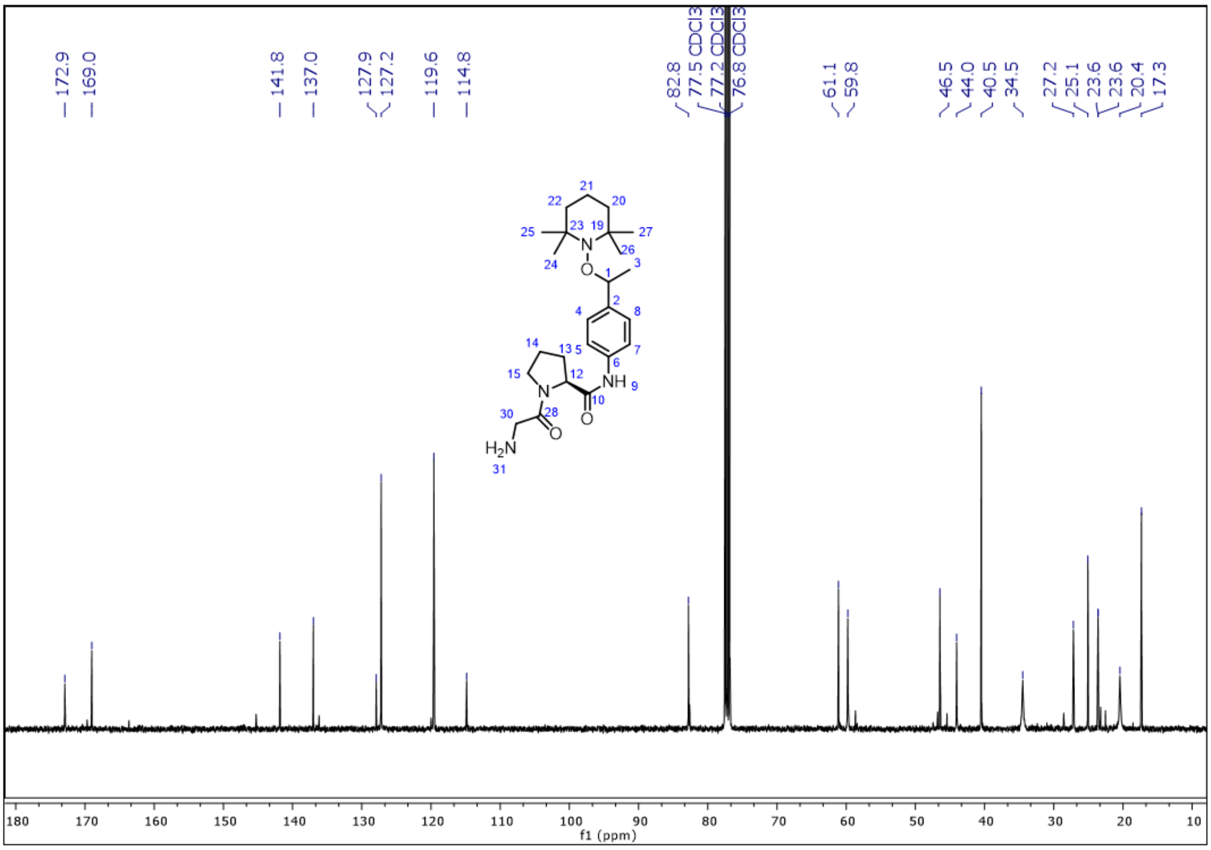

Vb: (2S)-1-(L-alanyl)-N-(4-(1-((2,2,6,6-tetramethylpiperidin-1-yl)oxy)ethyl)phenyl)pyrrolidine-2-carboxamide

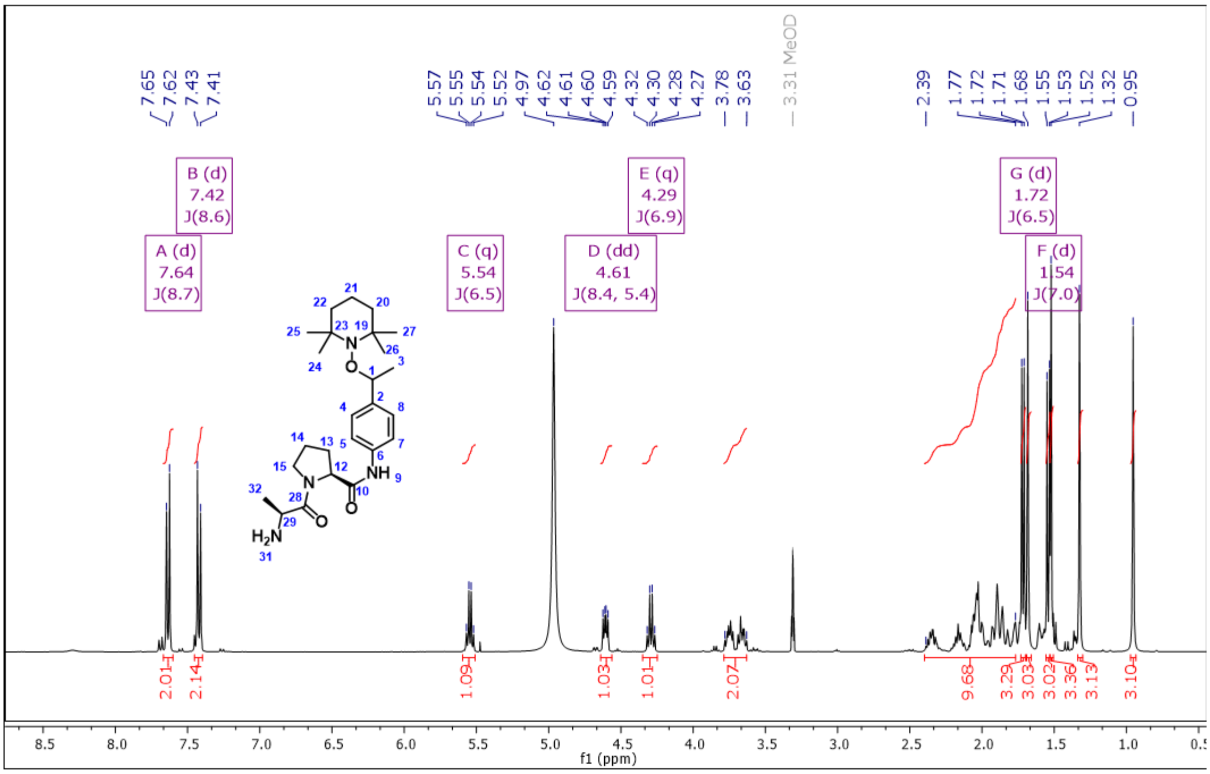

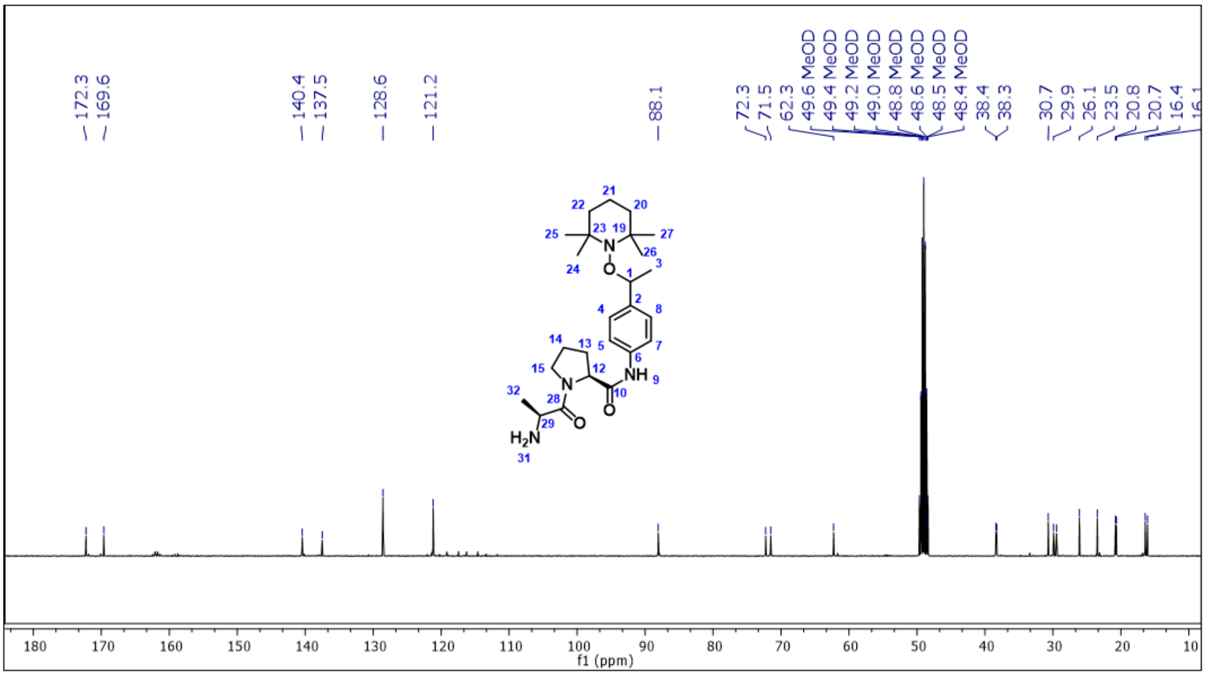

VIa: 4-oxo-4-((2-oxo-2-((2S)-2-((4-(1-((2,2,6,6-tetramethylpiperidin-1-yl)oxy)ethyl)phenyl) carbamoyl)pyrrolidin-1-yl)ethyl)amino)butanoic acid

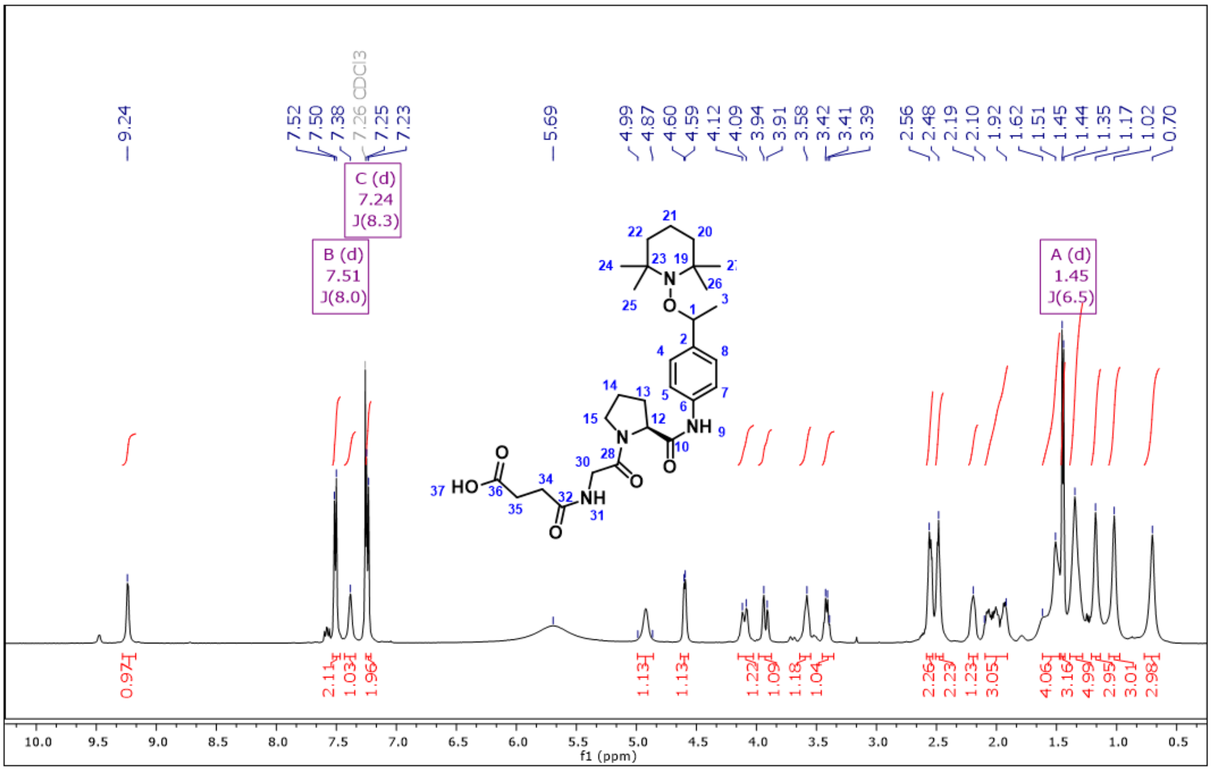

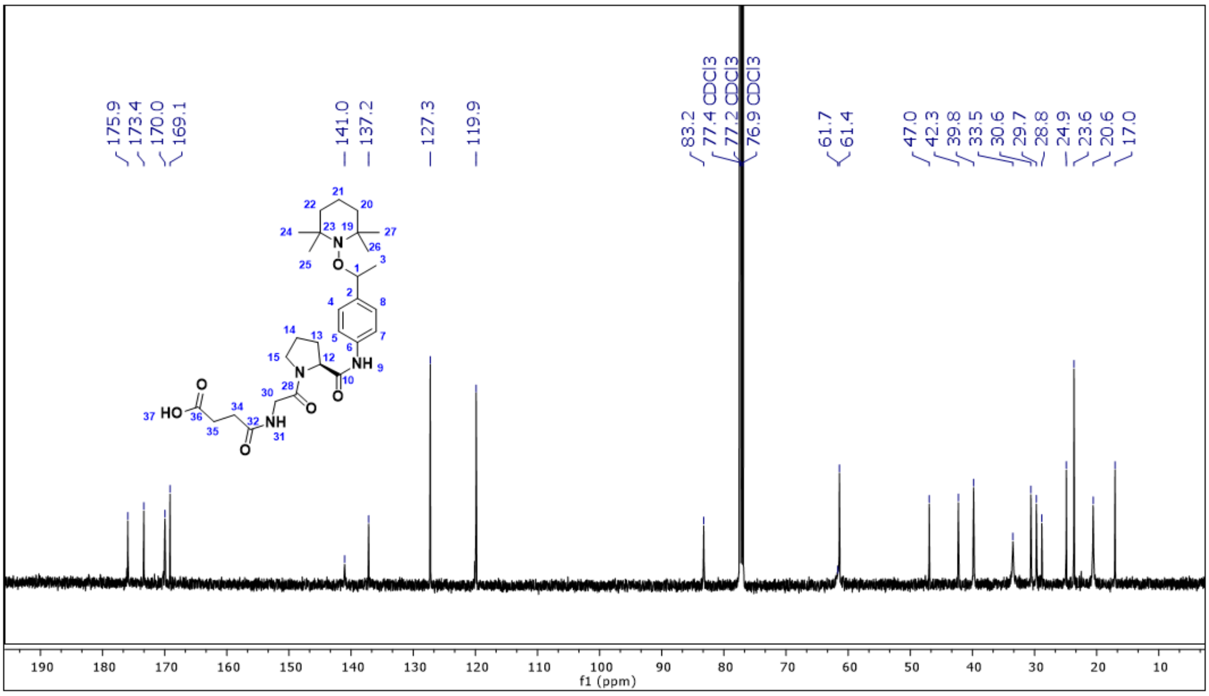

VIb: 4-oxo-4-(((2S)-1-oxo-1-((2S)-2-((4-(1-((2,2,6,6-tetramethylpiperidin-1-yl)oxy)ethyl)phenyl) carbamoyl)pyrrolidin-1-yl)propan-2-yl)amino)butanoic acid

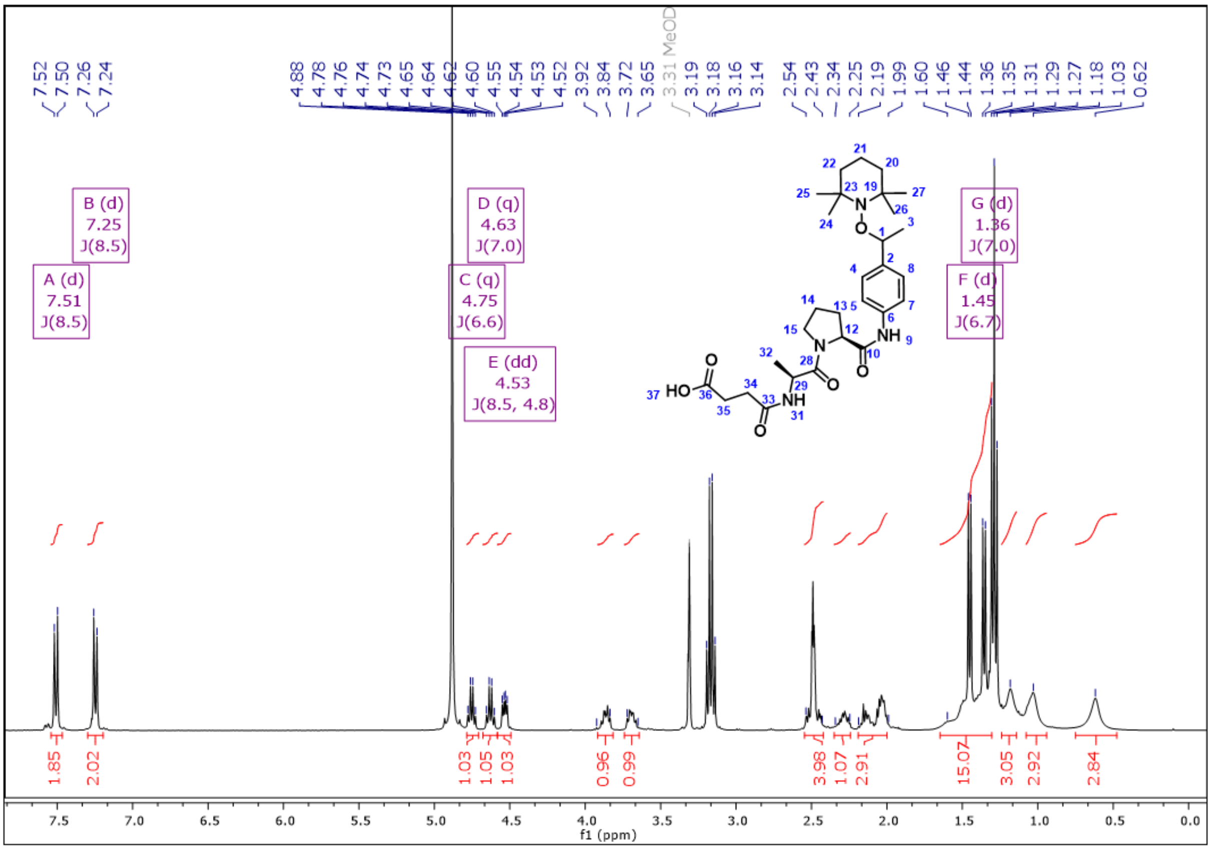

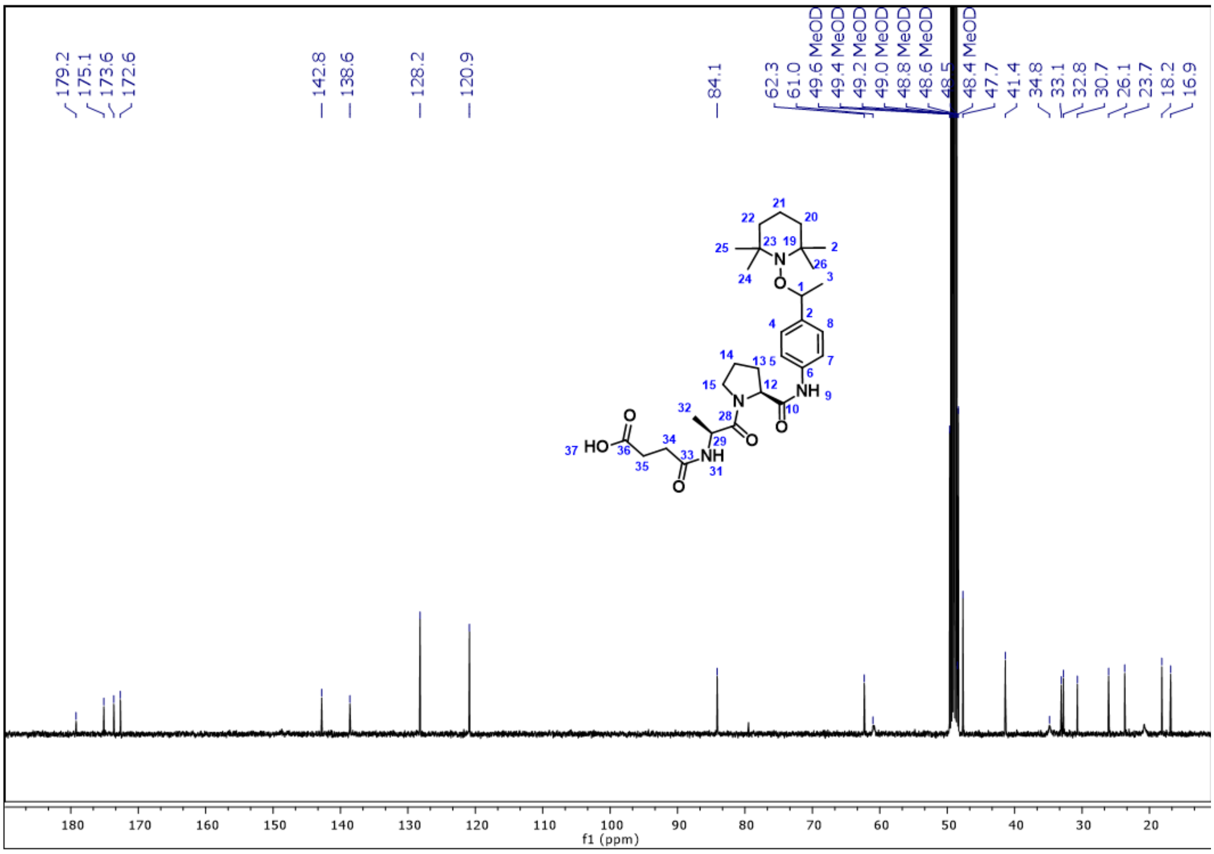

T1: tert-butyl (S)-2-((4-ethylphenyl)carbamoyl)pyrrolidine-1-carboxylate

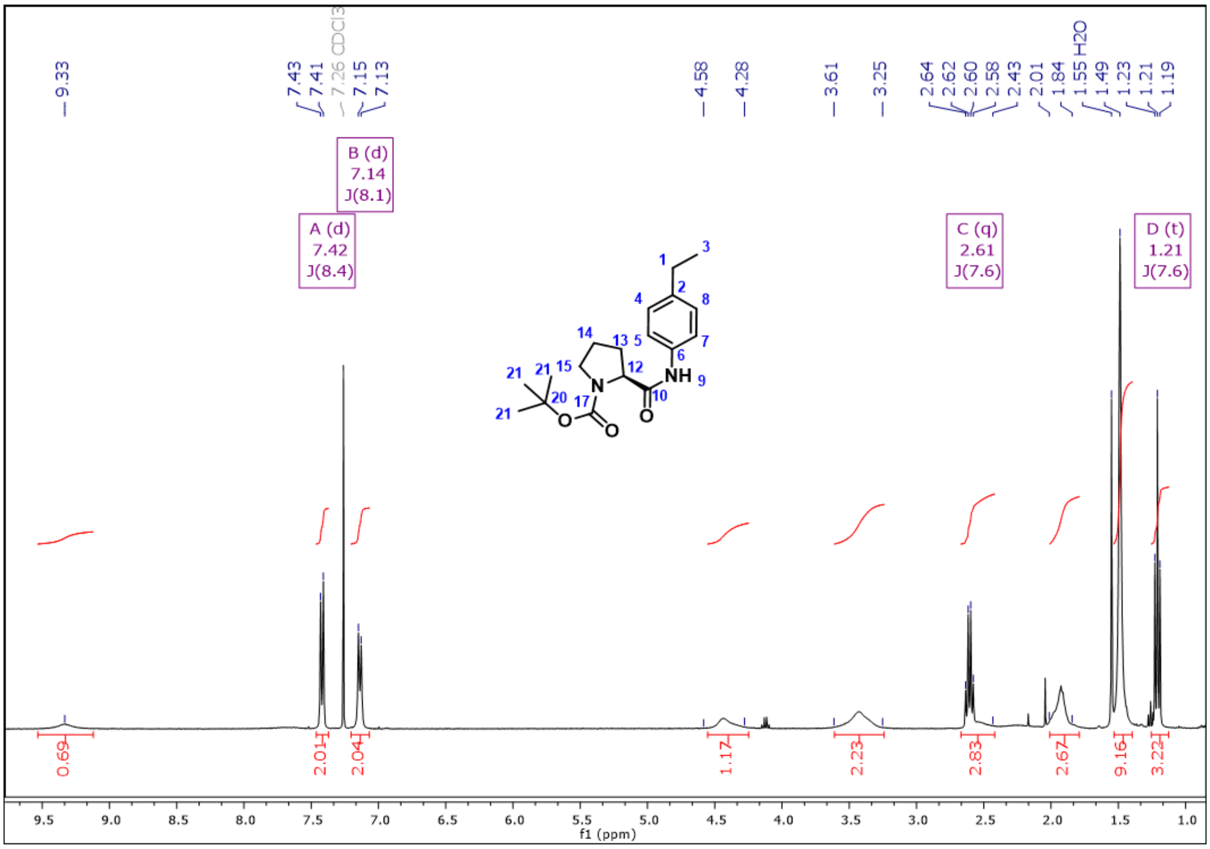

T2: (9H-fluoren-9-yl)methyl((S)-1-((S)-2-((4-ethylphenyl)carbamoyl)pyrrolidin-1-yl)-1-oxopropan-2-yl)carbamate

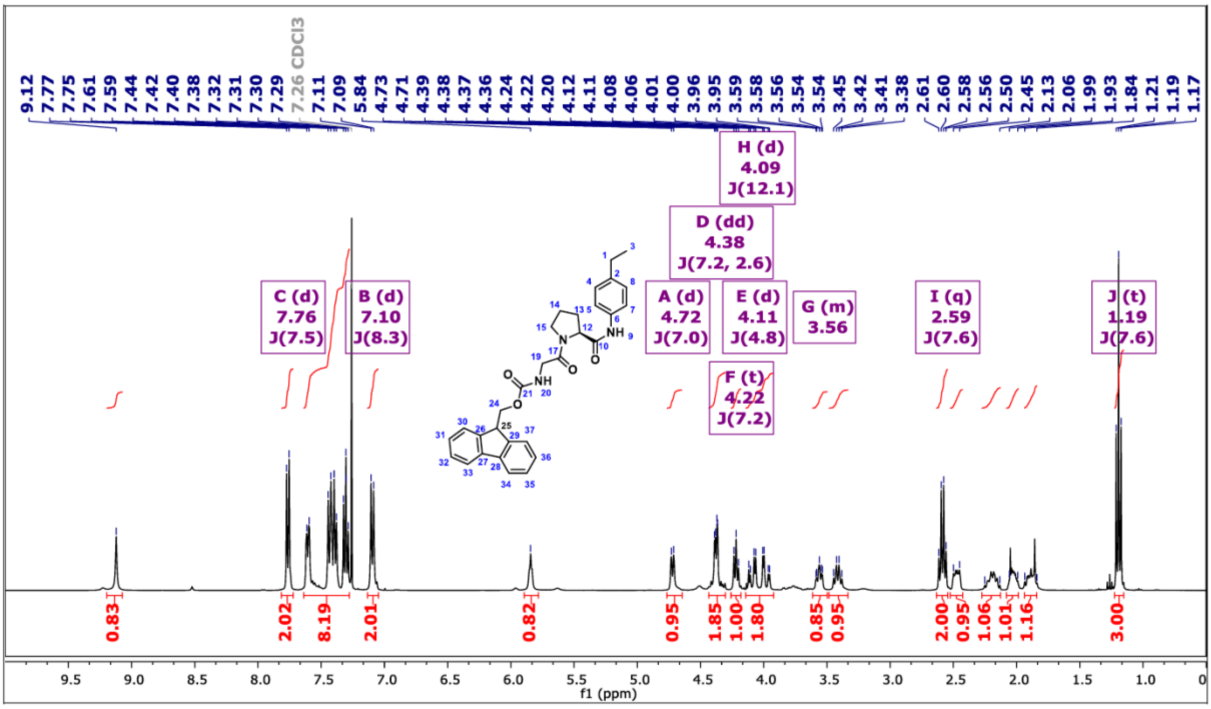

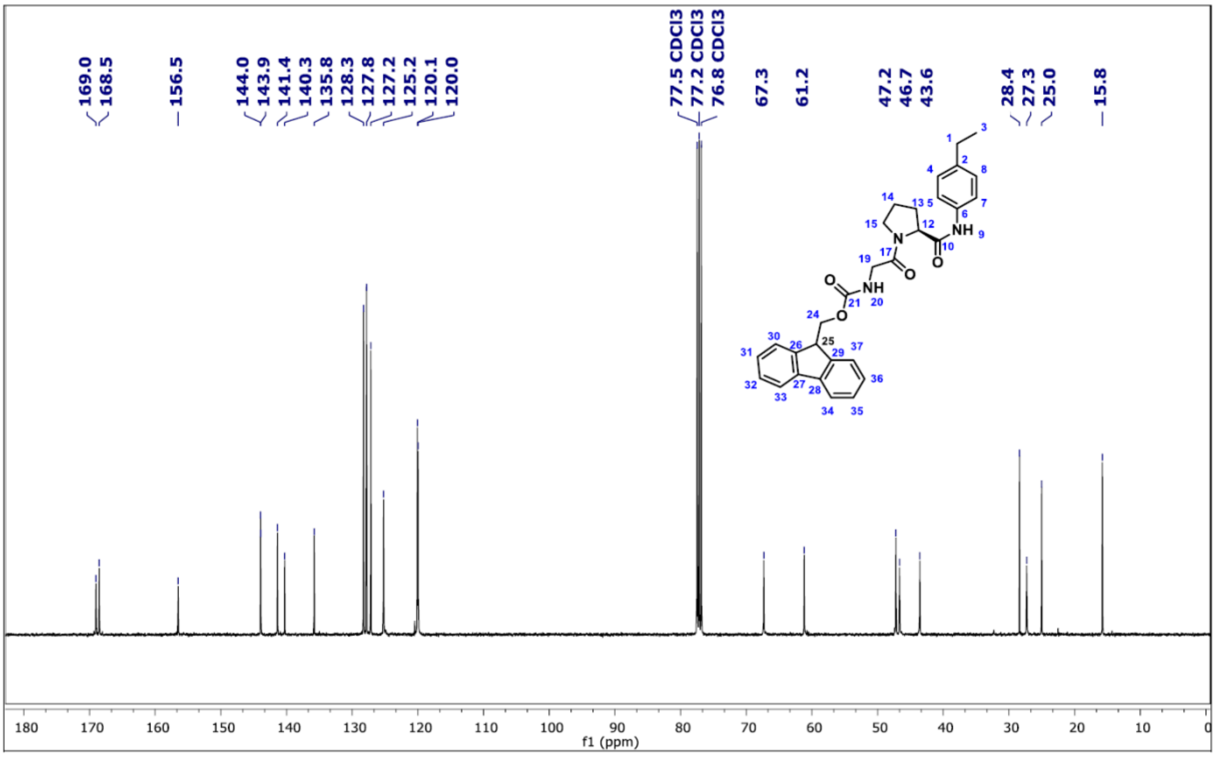

VIc: (S)-2-amino-1-((S)-2-(2-(4-ethylphenyl)acetyl)pyrrolidin-1-yl)propan-1-one

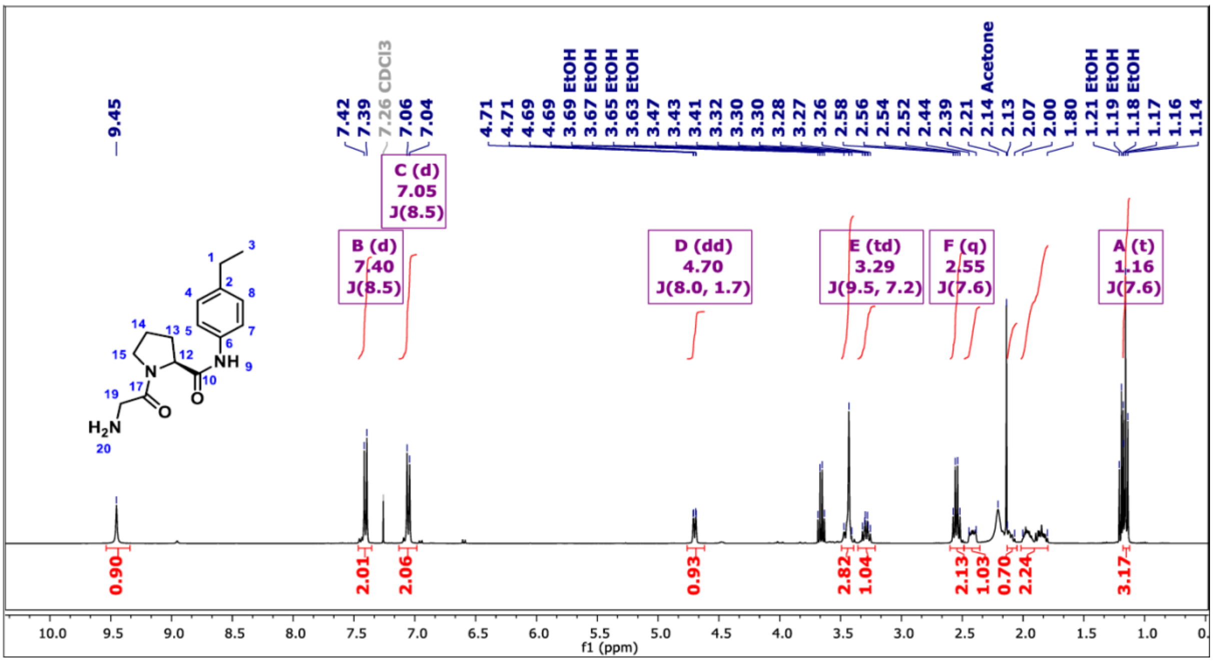

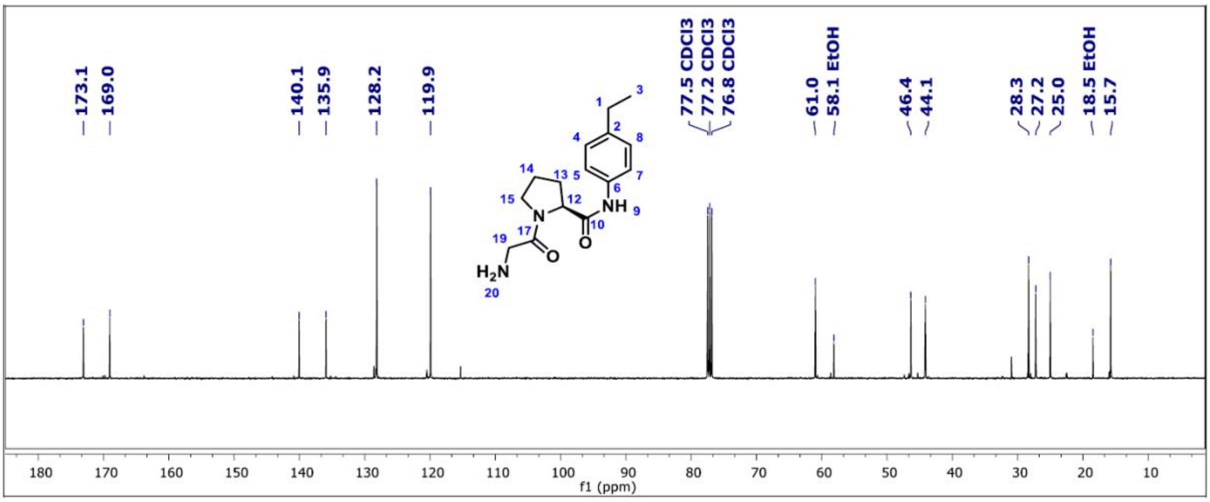

II. High-resolution mass spectrometry chromatograms

I: tert-butyl (S)-2-((4-vinylphenyl)carbamoyl)pyrrolidine-1-carboxylate

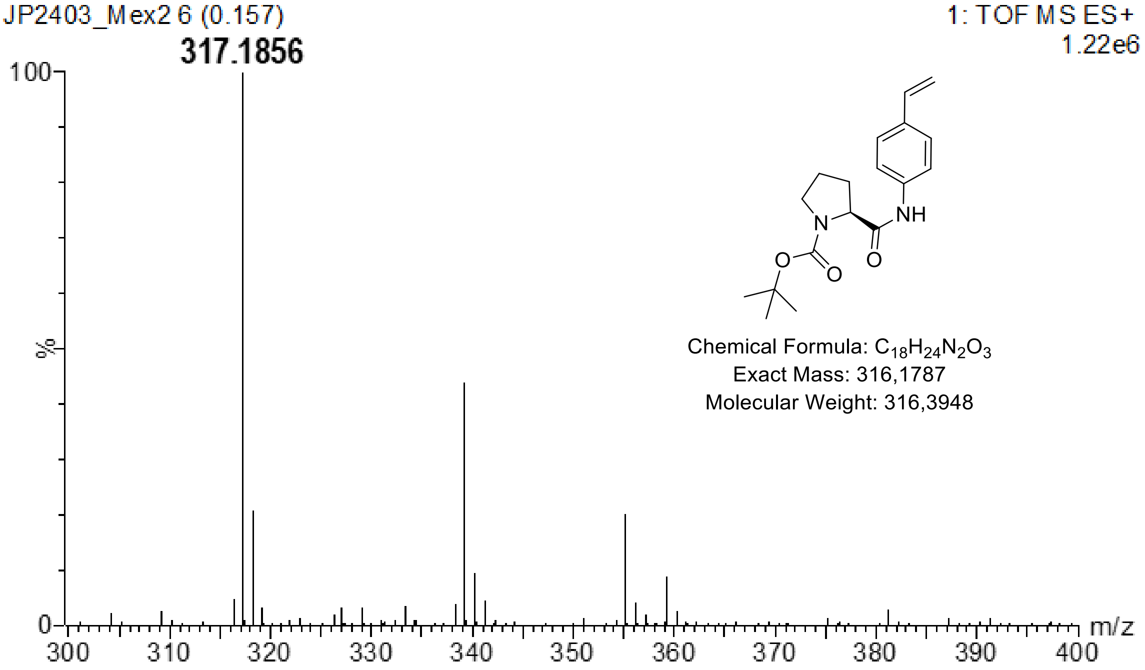

II: tert-butyl (2S)-2-((4-(1-((2,2,6,6-tetramethylpiperidin-1-yl)oxy)ethyl)phenyl)carbamoyl) pyrrolidine-1-carboxylate

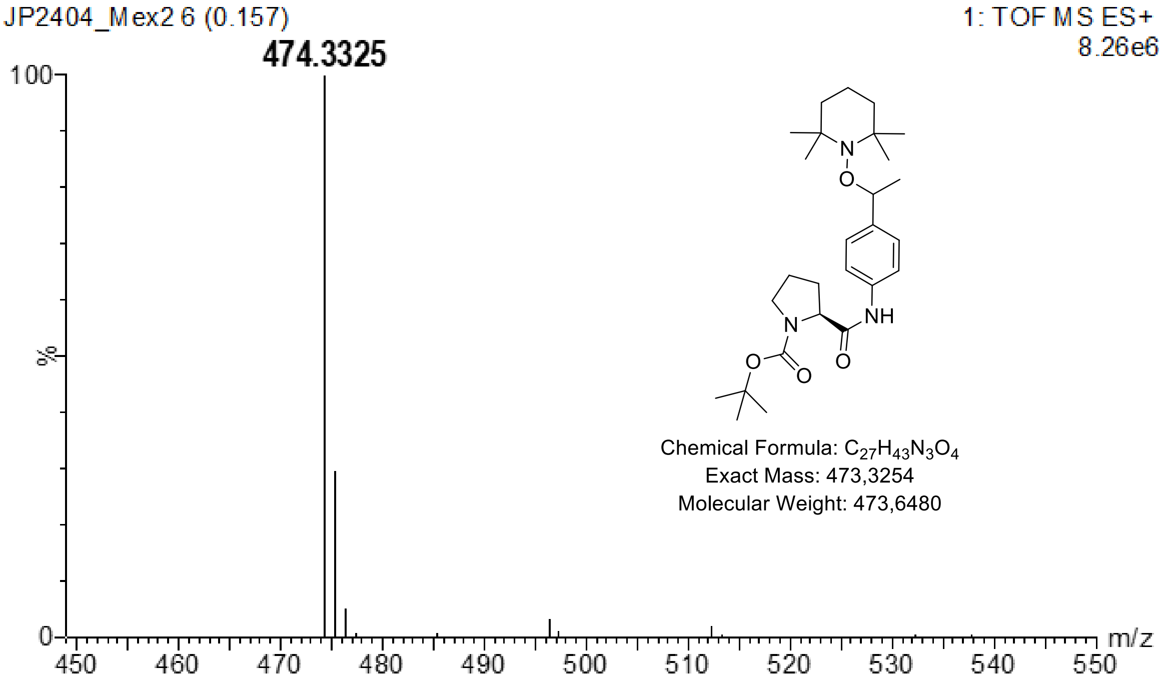

III: (2S)-2-((4-(1-((2,2,6,6-tetramethylpiperidin-1-yl)oxy)ethyl)phenyl)carbamoyl)pyrrolidin-1-ium

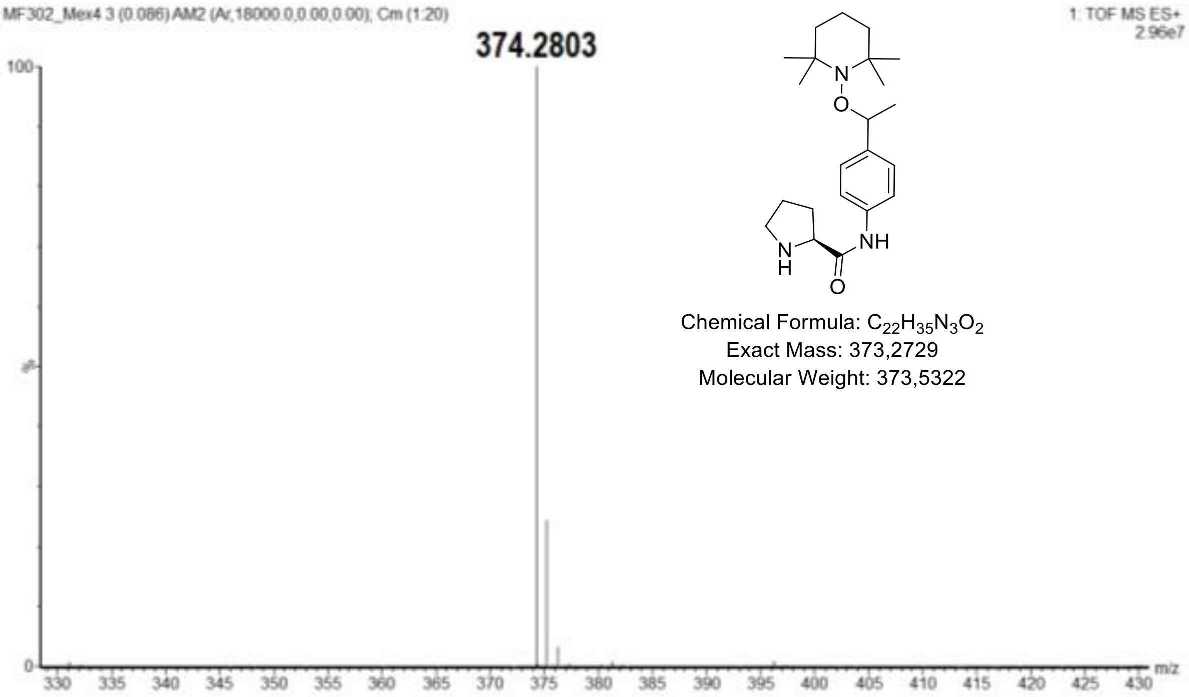

IVa: (9H-fluoren-9-yl)methyl (2-oxo-2-((2S)-2-((4-(1-((2,2,6,6-tetramethylpiperidin-1-yl)oxy)ethyl) phenyl)carbamoyl)pyrrolidin-1-yl)ethyl)carbamate

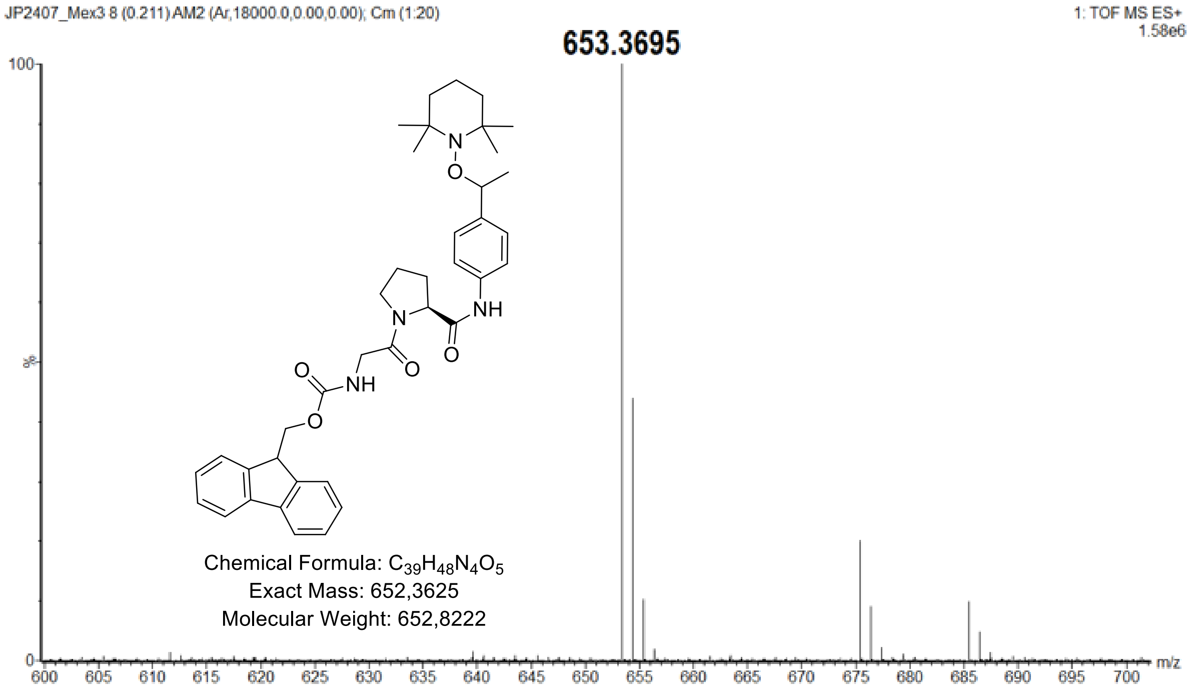

Va: (2S)-1-glycyl-N-(4-(1-((2,2,6,6-tetramethylpiperidin-1-yl)oxy)ethyl)phenyl)pyrrolidine-2-carboxamide

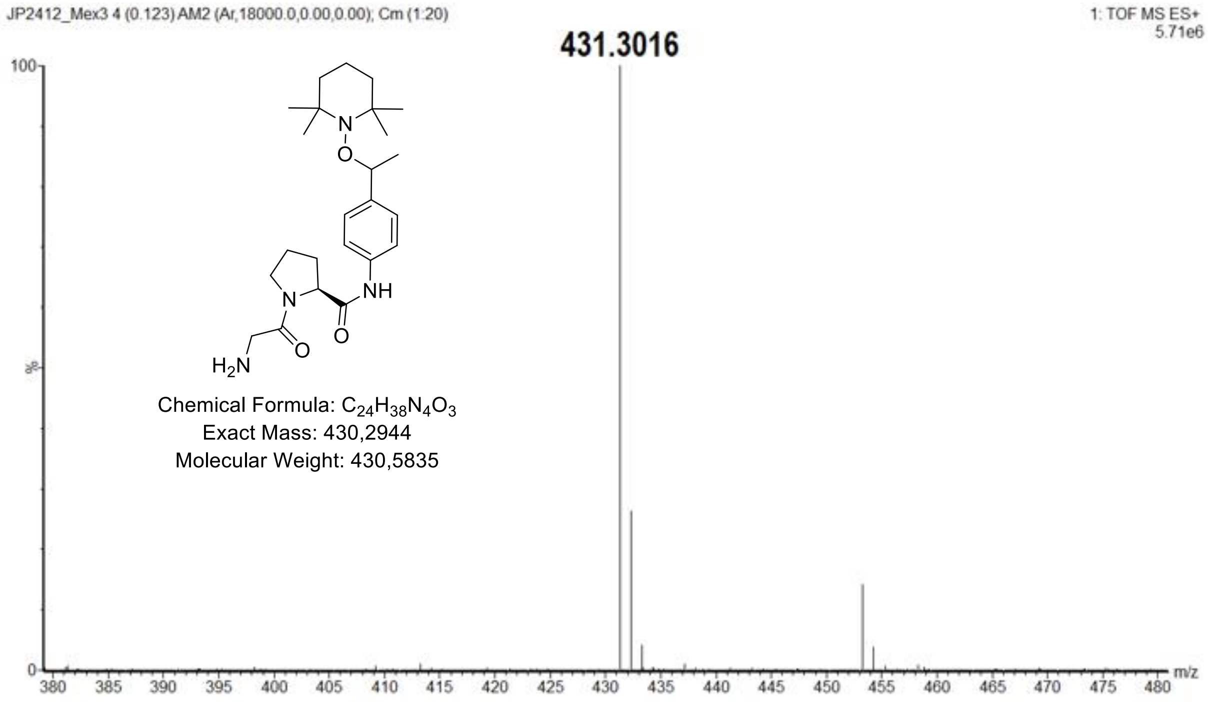

VIa: 4-oxo-4-((2-oxo-2-((2S)-2-((4-(1-((2,2,6,6-tetramethylpiperidin-1-yl)oxy)ethyl)phenyl) carbamoyl)pyrrolidin-1-yl)ethyl)amino)butanoic acid

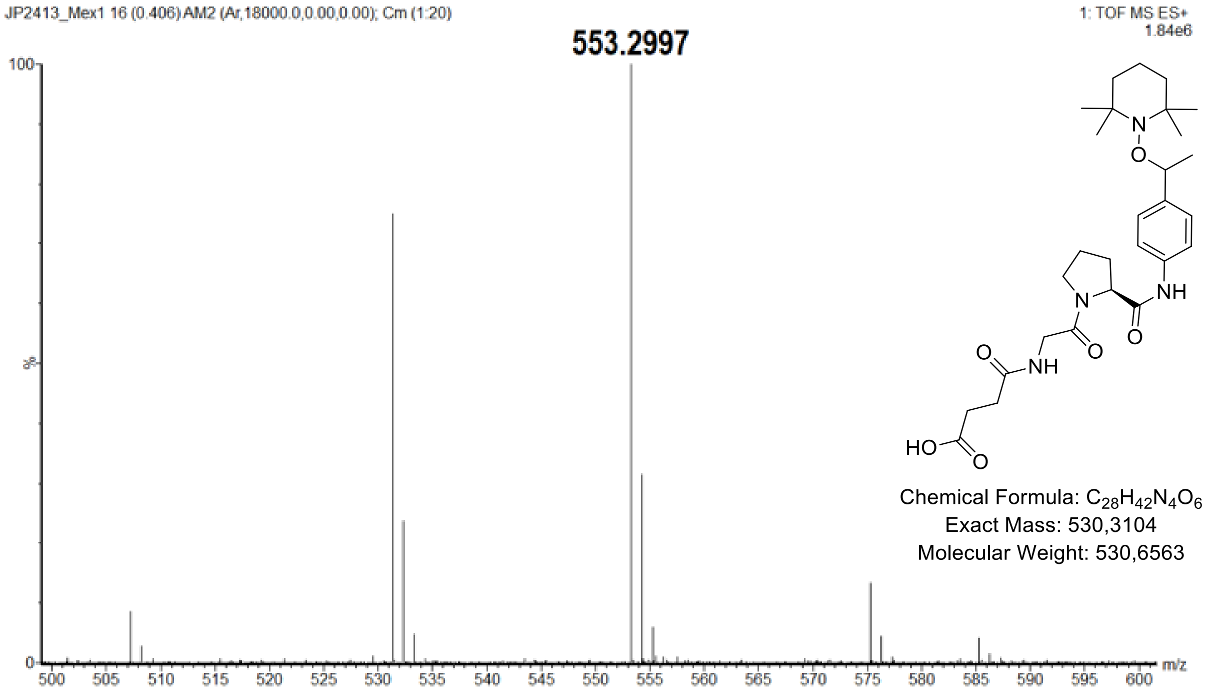

IVb: (9H-fluoren-9-yl)methyl ((2S)-1-oxo-1-((2S)-2-((4-(1-((2,2,6,6-tetramethylpiperidin-1-yl)oxy) ethyl)phenyl)carbamoyl)pyrrolidin-1-yl)propan-2-yl)carbamate

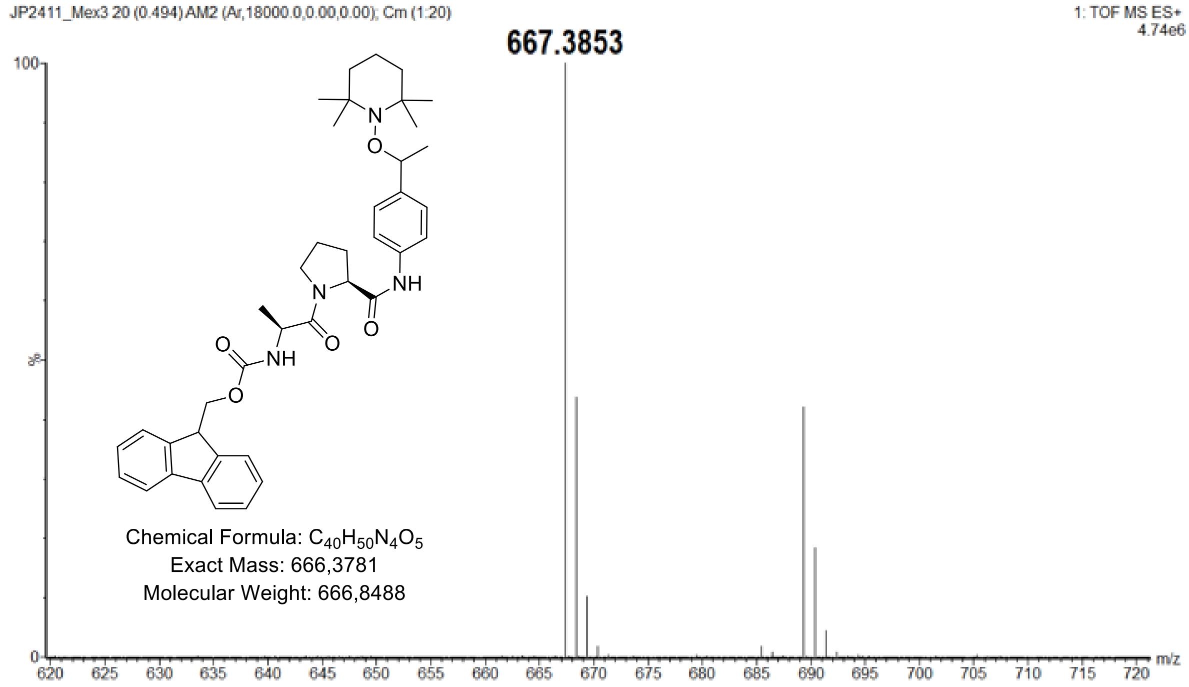

Vb: (2S)-1-(L-alanyl)-N-(4-(1-((2,2,6,6-tetramethylpiperidin-1-yl)oxy)ethyl)phenyl)pyrrolidine-2-carboxamide

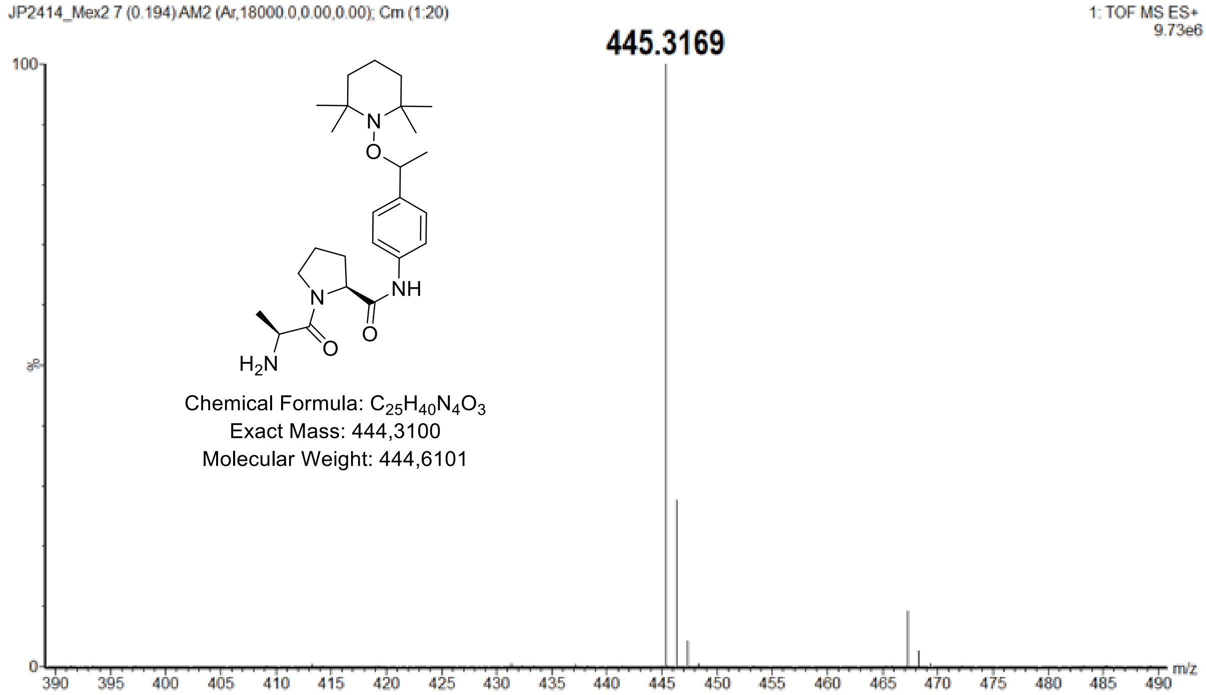

VIb: 4-oxo-4-(((2S)-1-oxo-1-((2S)-2-((4-(1-((2,2,6,6-tetramethylpiperidin-1-yl)oxy)ethyl)phenyl) carbamoyl)pyrrolidin-1-yl)propan-2-yl)amino)butanoic acid

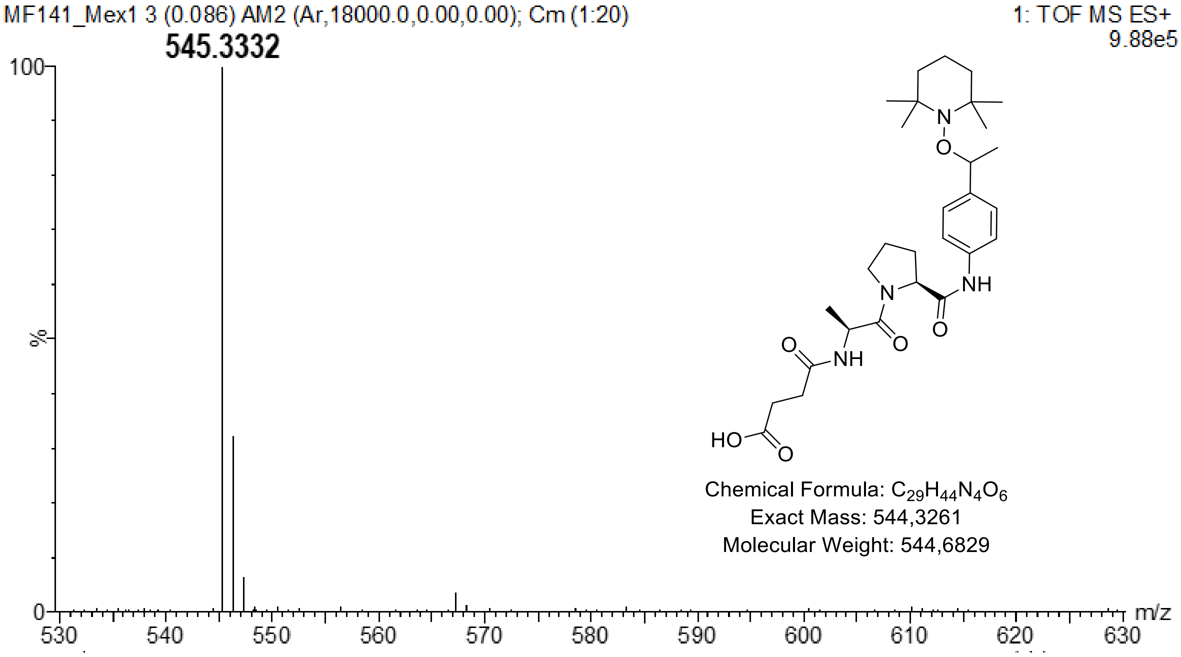

T2: (9H-fluoren-9-yl)methyl((S)-1-((S)-2-((4-ethylphenyl)carbamoyl)pyrrolidin-1-yl)-1-oxopropan-2-yl)carbamate

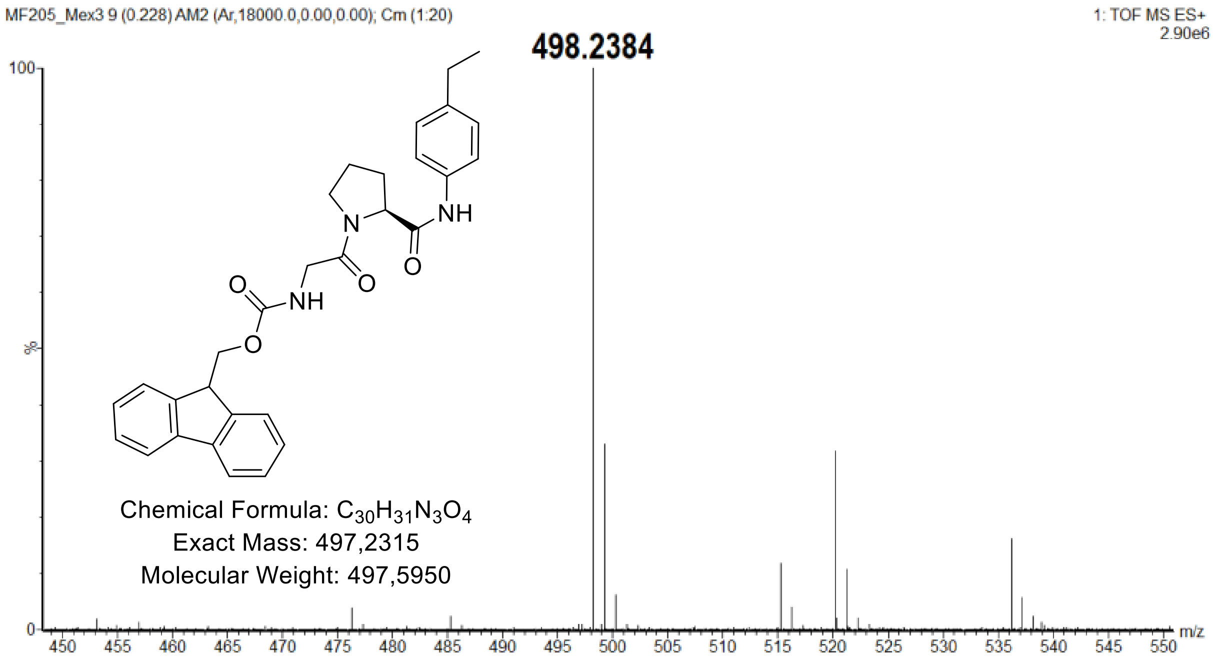

VIc: (S)-2-amino-1-((S)-2-(2-(4-ethylphenyl)acetyl)pyrrolidin-1-yl)propan-1-one

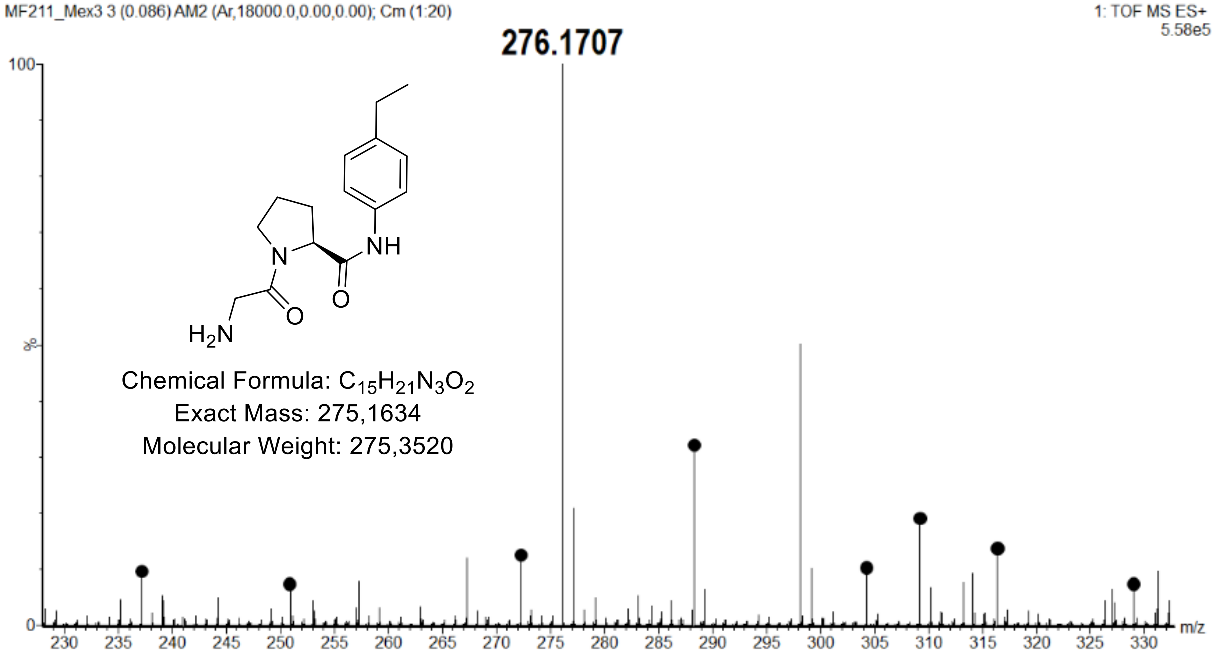

SUPPLEMENTARY FIGURE 3

Kinetics of TEMPO release (concentration over time) for Va at 132 °C

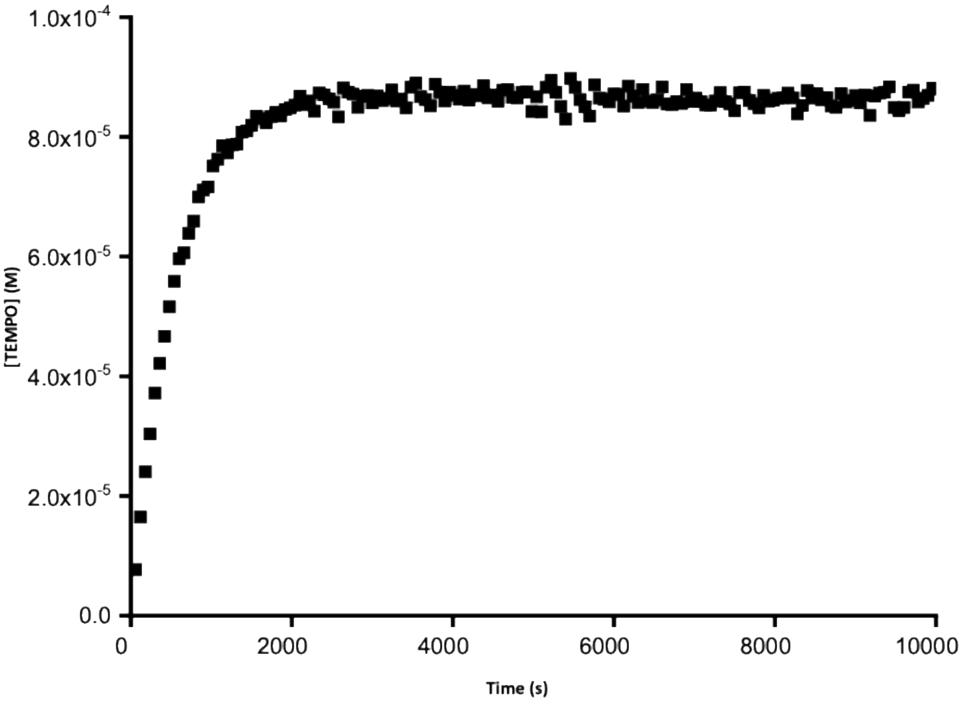

SUPPLEMENTARY FIGURE 4

Enzymatic activation followed by homolysis of the alkoxyamine

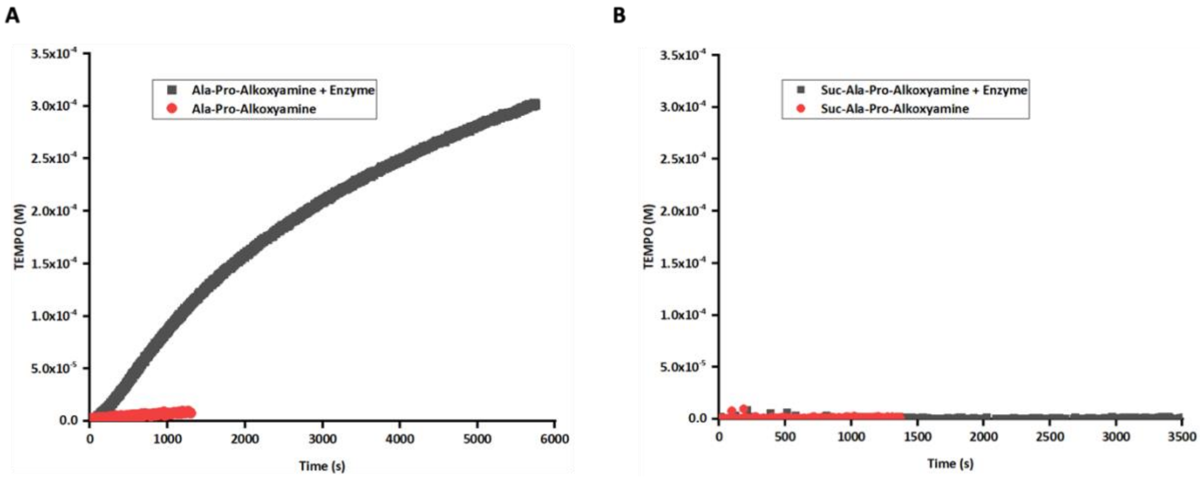

SUPPLEMENTARY FIGURE 5

Alkoxyamine Va alters the epithelial monolayer integrity.

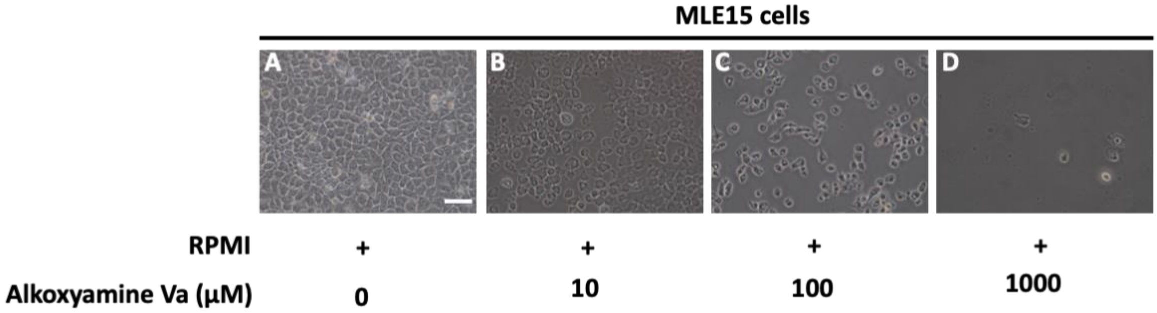

Supplementary figure 5. Alkoxyamine Va alters epithelial monolayer integrity. Confluent cultured MLE15 cells were cultured alone or the presence of various concentrations of alkoxyamine Va. A-D/ Representative phase contrast images of untreated (A) or Alkoxyamine Va-exposed (B-D) MLE15 cells. A/ Intact monolayer of untreated cells. B-D/ Cell exposure to gradual increase of alkoxyamine Va concentrations (10, 100, 1000 µM) resulted in progressive disruption of cell-cell adhesion and considerable loss of integrity and floating of cells. Scale bar, 40 µm. Experiments were repeated three times with similar observations.

SUPPLEMENTARY TABLE1

EPR kinetic figures of homolysis rate constants (k_d), activation energies (E_a) and half-life times ($t_{1/2}$):

Molecule	Solvent	T (°C)[a]	k_d (s^{-1})[b,c]	E_a ($kJ \cdot mol^{-1}$)[d]	$t_{1/2}$(37 °C) (days)
2	*t*-BuPh	134	$4.8 \cdot 10^{-4}$	137.8	5395
3	*t*-BuPh/DCM 99:1	130	$5.9 \cdot 10^{-4}$	135.9	2554
4a	*t*-BuPh	135	$3.4 \cdot 10^{-4}$	139.5	10651
5a	*t*-BuPh	122	$3.8 \cdot 10^{-4}$	134.6	1537
5a	H_2O/1-PrOH 99:1	87	$2.8 \cdot 10^{-5}$	130.4	308
6a	*t*-BuPh/DCM 99:1	132	$1.1 \cdot 10^{-3}$	134.5	1519
6a	H_2O/1-PrOH 99:1	86	$4.9 \cdot 10^{-4}$	121.6	10
4b	*t*-BuPh	134	$3.9 \cdot 10^{-4}$	138.7	9117
5b	*t*-BuPh	133	$8.8 \cdot 10^{-4}$	135.6	2262
5b	H_2O/1-PrOH 99:1	90	$5.6 \cdot 10^{-5}$	129.7	230
6b	*t*-BuPh/DCM 99:1	133	$6.3 \cdot 10^{-4}$	136.8	3632
6b	H_2O/1-PrOH 99:1	89	$5.5 \cdot 10^{-5}$	129.3	199

[a] Error is ± 1 °C. [b] Determined using equation 1. Statistical error < 5%. [c] Values measured for a mixture of diastereoisomers. [d] Activation energies were estimated using a frequency factor A of $2.4 \cdot 10^{14}$ s^{-1}, as given in equation 2. Errors in activation energies are less than 1-2 $kJ \cdot mol^{-1}$.

Equation 1. Rate constant determination $\ln\left(\frac{[nitroxide]_{\infty} - [nitroxide]_{t}}{[nitroxide]_{\infty}}\right) = -k_{d} \cdot t$

Equation 2. Arrhenius law $E_a = -\mathrm{R} \cdot T \cdot \ln\left(\frac{k_d}{\mathrm{A}}\right)$
